# A Little CFTR Goes a Long Way: CFTR-Dependent Sweat Secretion from G551D and R117H-5T Cystic Fibrosis Subjects Taking Ivacaftor

**DOI:** 10.1371/journal.pone.0088564

**Published:** 2014-02-10

**Authors:** Jessica E. Char, Marlene H. Wolfe, Hyung-ju Cho, Il-Ho Park, Jin Hyeok Jeong, Eric Frisbee, Colleen Dunn, Zoe Davies, Carlos Milla, Richard B. Moss, Ewart A. C. Thomas, Jeffrey J. Wine

**Affiliations:** 1 Cystic Fibrosis Research Laboratory, Stanford University, Stanford, California, United States of America; 2 Department of Pediatrics, Stanford University School of Medicine, Stanford, California, United States of America; 3 Department of Psychology, Stanford University, Stanford, California, United States of America; Tor Vergata University of Rome, Italy

## Abstract

To determine if oral dosing with the CFTR-potentiator ivacaftor (VX-770, Kalydeco) improves CFTR-dependent sweating in CF subjects carrying G551D or R117H-5T mutations, we optically measured sweat secretion from 32–143 individually identified glands in each of 8 CF subjects; 6 F508del/G551D, one G551D/R117H-5T, and one I507del/R117H-5T. Two subjects were tested only (−) ivacaftor, 3 only (+) ivacaftor and 3 (+/−) ivacaftor (1–5 tests per condition). The total number of gland measurements was 852 (−) ivacaftor and 906 (+) ivacaftor. A healthy control was tested 4 times (51 glands). For each gland we measured both CFTR-independent (M-sweat) and CFTR-dependent (C-sweat); C-sweat was stimulated with a β-adrenergic cocktail that elevated [cAMP]_i_ while blocking muscarinic receptors. Absent ivacaftor, almost all CF glands produced M-sweat on all tests, but only 1/593 glands produced C-sweat (10 tests, 5 subjects). By contrast, 6/6 subjects (113/342 glands) produced C-sweat in the (+) ivacaftor condition, but with large inter-subject differences; 3–74% of glands responded with C/M sweat ratios 0.04%–2.57% of the average WT ratio of 0.265. Sweat volume losses cause proportionally larger underestimates of CFTR function at lower sweat rates. The losses were reduced by measuring C/M ratios in 12 glands from each subject that had the highest M-sweat rates. Remaining losses were estimated from single channel data and used to correct the C/M ratios, giving estimates of CFTR function (+) ivacaftor  = 1.6%–7.7% of the WT average. These estimates are in accord with single channel data and transcript analysis, and suggest that significant clinical benefit can be produced by low levels of CFTR function.

## Introduction

Genetic mutations that reduce CFTR-mediated anion conductance (G_anion_ = n*P_O_*γ ) cause cystic fibrosis (CF). Over 1,000 mutations are known or predicted to reduce CFTR channel number (n), open probability (*P_O_*), and/or conductance (γ), and 127 of these have been identified as disease-causing mutations [1]. A complex set of interacting pathophysiological consequences follows large reductions in CFTR function, most resulting from defective ion and fluid transport. The most efficient way to stop this cascade is to correct the defective gene or protein. Ivacaftor (VX-770, Kalydeco) increases *P_O_* in a wide variety of CFTR mutations as well as WT CFTR [2]. VX-770 was shown to improve the function of G551D in cultured cells [3], and patients having at least one G551D mutation treated with oral ivacaftor showed marked clinical improvement [4], [5], leading to FDA approval of ivacaftor for use in G551D patients. Recently, ivacaftor was shown to improve CFTR-dependent ion transport in human airway epithelial cells carrying an R117H mutation [6], and trials are now underway to determine if ivacaftor will be clinically beneficial for patients with R117H-5T mutations.

Most CFTR mutations are rare, and marked phenotypic differences can occur in patients having the same mutations [7], [8], [9]. Therefore, n-of-1 studies are informative. With that in mind, a bioassay was developed that compares the volume of CFTR-independent and CFTR-dependent sweat secretion gland-by-gland for ∼50 individually identified glands within each subject [10]. On the premise that the therapeutics of interest will act directly on CFTR, we treated individual glands as the units of analysis, providing sufficient statistical power to determine efficacy for individual subjects in n-of-1 studies.

The assay takes advantage of two parallel pathways for sweat secretion: a CFTR-independent, cholinergic pathway stimulated with methacholine (‘M-sweat’) and a β-adrenergic pathway that is CFTR-dependent (‘C-sweat’) [10]. When C-sweating is expressed as a function of M-sweating, the assay gives a near-linear readout of CFTR function over a wide range: i.e. the C-sweat/M-sweat ratio for carriers of one CFTR mutation is 50% that of non-CF controls, while the ratio for CF subjects is zero. However, at very low levels of CFTR function the C-sweat assay departs from linearity and becomes less sensitive: at present it doesn’t detect C-sweating in most pancreatic sufficient (PS) CF subjects, who are known to have some residual CFTR function [10]. The sweat volume bioassay is thus complementary to the sweat chloride test, which does discriminate between CF patients based on their pancreatic status, but which can’t discriminate CF heterozygotes from WT subjects [10]. Both tests benefit by using an organ that is unaffected by the progressive, destructive changes that occur in many other CFTR-expressing organs [11].

Given the assay's present inability to discriminate between PI and PS subjects, it was important to determine if it could detect functional efficacy of ivacaftor in CF subjects carrying the G551D mutation, where its clinical efficacy has already been demonstrated [4], [5]. Therefore, in the present study we measured the efficacy of ivacaftor in 4 individuals of G551D/F508del genotype. In addition, we wanted to use the assay with patients having mutations for which clinical efficacy of ivacaftor is presently unknown. Therefore, we also used the assay to measure the efficacy of ivacaftor in two CF subjects with R117H-5T mutations.

## Materials and Methods

### Subjects

The study was approved by the Institutional Review Board of Stanford University. After written informed consent, six adults with genotypes G551D/F508del, one with G551D/R117H-5T and one with I507del/R117H-5T were enrolled, along with a healthy control. All CF subjects were classified as having cystic fibrosis by the Stanford Cystic Fibrosis Center on the basis of elevated sweat chloride levels, CFTR mutations, and clinical indications. The healthy control subject was genotyped by the Molecular Pathology Laboratory, Stanford University Medical Center and was free of any CFTR mutations and had (TG)12-7T and (TG)10-9T.

Two subjects were tested only off drug, three only on drug, and two were tested both off and on. There were no adverse events. Seven subjects had been enrolled in multi-center studies of ivacaftor and were studied either during the open label period, during the month prior to the start of open label, or both. One G551D subject who was not enrolled in an ivacaftor trial was studied off drug prior to FDA approval and then on-drug after starting ivacaftor as prescribed by his physician.

### Ratiometric measurement of sweat secretion from identified individual glands

We used the single gland, ratiometric, optical assay for CFTR secretory function as described by Wine *et al.* [10]. In brief, a specific region of skin on the volar forearm was sequentially injected intradermally with methacholine to stimulate CFTR-independent sweating (M-sweat) and then with a cocktail of agents that blocked M-sweating and produced CFTR-dependent sweating (C-sweat). Bubbles of sweat from single glands were captured in an oil layer, visualized by oblique lighting or dye-partioning, and digitally imaged at 30 sec intervals. The individual glands were identified by location as follows: a region of interest (ROI) was defined by counting bubbles of M-sweat starting at the center of the field and spiraling outward until ∼50 individual glands were numbered. For each identified gland the increases in M- and C-sweat volumes over time were measured. Plots of volume vs. time were made at 5-6 min intervals for selected glands to latencies and rates of volume increase. Plots correlating M- and C-sweat volumes were based on M-sweat volumes at the end of 15 min, and C-sweat volumes/2 at the end of 30 min. In the plots of M- and C-sweat correlations, each point represents the average volume for a single identified gland based on all trials where the gland was measured.

To provide a common, meaningful basis of comparison among the CF subjects, we obtained a mean C/M ratio of 0.2651 from testing six healthy controls subjects [10], set that value  = 100%, and expressed the responses of the CF subjects with reference to it. Proportionality between CFTR-dependent and CFTR-independent secretory function, i.e., the constancy of the C/M ratio across glands, is compromised by sweat volume losses via ductal absorption and physical capacitance [10], as well as by finite losses into the oil from very small bubbles in spite of extensive water saturation of the oil (P.M. Quinton, personal communication). Indeed, the C/M values showed a positive relation to M-sweat rates, consistent with proportionally less sweat volume loss in the faster secreting glands. To take advantage of this relationship for each subject, we plotted the C/M ratio for the 12 highest M-sweat secreting glands, in which sweat loss is much reduced and the C/M ratio is expected to be relatively constant. A further correction factor was obtained by using single channel measures of *P_O_* and γ for R117H [12] and mRNA transcript measurements of n [13] to provide an independent estimate of unpotentiated R117H-5T channel function. We then subtracted the C/M measure from the predicted single channel value to obtain an estimate of the amount of fluid loss, and added this back for a final, best estimate of CFTR function (see discussion). For G551D, our corrected C/M values were compared with single channel measures made (±) ivacaftor [14], [15].

**Figure 1:**
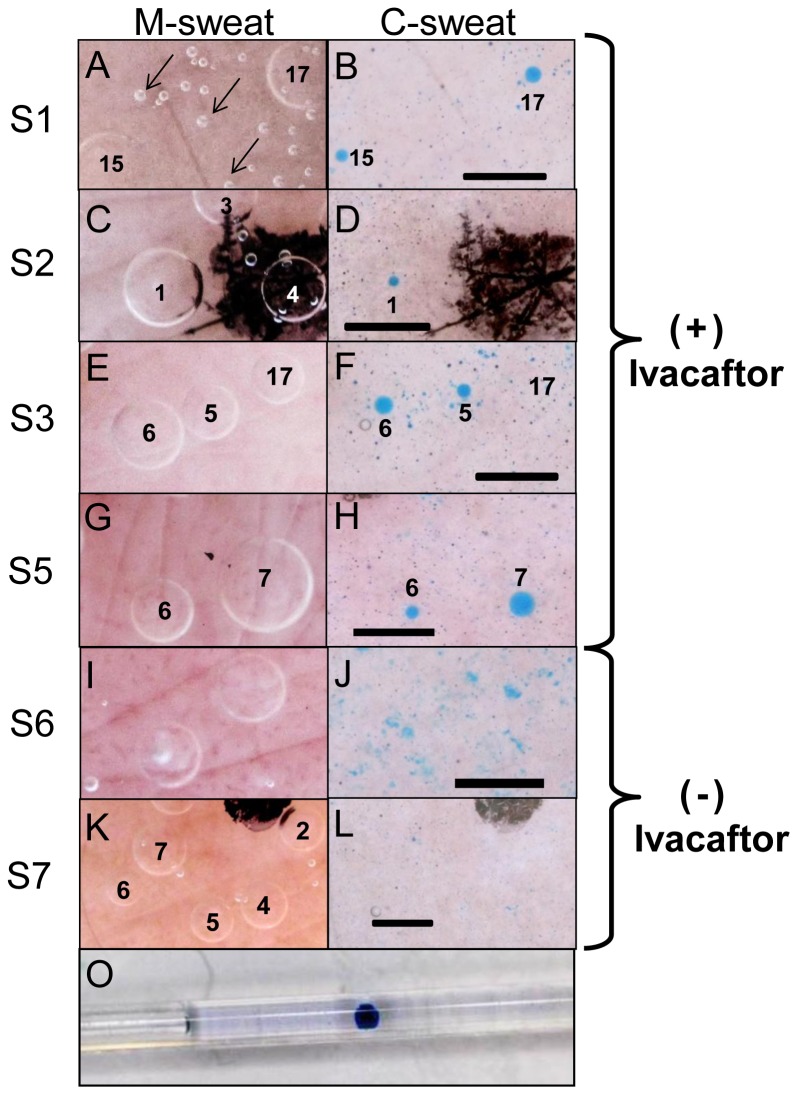
CFTR independent (M-sweat) and CFTR dependent (C-sweat) secretion in G551D CF subjects. Each image shows a small region (∼1.5 mm^2^) of the stimulated field of sweat glands (full imaged field is 0.63 cm^2^) for each subject. Images for S1-3 and S5 were (+) ivacaftor and S6, 7 (−) ivacaftor. A portion of each field was selected to show glands that responded (or not) to the β-adrenergic cocktail (right column). (A-B). S1, male. Arrows in (A) point to air bubbles in the oil. (C-D) S2, female. (D) Shows the single sweat gland that responded to the β-adrenergic stimulus in this experiment. The dark stain is an ink spot on the freckle used for image registration. (E-F) S3, female. This subject had high background staining. (G-H) S5, male. (I-J) S6, female, (-) ivacaftor. This subject showed the most background staining of any subject in this series. (K-L) S7, female, (−) ivacaftor. (O) C-sweat collected in 2 µl constant bore capillary from S1 (+) ivacaftor. Calibration bars  = 0.5 mm.

The small volumes of C-sweat observed for the CF subjects made us alert to possible artifacts. In addition to other criteria [10], we also used pipets to collect C-sweat from three CF subjects after optically recording secretion in the presence of ivacaftor. The small amounts collected were in accord with the optical measurements. We tried but failed to collect any C-sweat from CF subjects (−) ivacaftor. Skin staining seen in some experiments (−) ivacaftor (e.g. **Fig. 1L**) had no detectable volume and could not be dislodged by the collecting pipet. Such staining was seen variably in 6 experiments done prior to 2/13/2012 when it was eliminated by a rinse procedure between the M-sweat and C-sweat trials. All (+) ivacaftor trials were done with minimized background and hence had reduced chances of false positives, but extremely low levels of secretion might have been missed in 3 (−) ivacaftor tests done before the rinse procedure.

### Statistical Analysis

Single, identified sweat glands were the units of analysis for the within-subject comparisons in our “n-of-1” analyses [10]. Paired t-tests on log transformed data, Pearson r, ANOVA, and mixed models regression with lmer() from the lme4 package in R (see methods, ref [10]) were used as appropriate.

The ratio of the average rates of M- and C-sweat produced per gland (averaged over 15 min for M-sweat and 30 min for C-sweat) is the main index of sweat production, and we use it here to estimate the proportion of CFTR function in various conditions relative to a sample of healthy controls. However, for CF subjects, virtually no glands produced C-sweat in the (-) ivacaftor condition, and the majority still failed to produce measurable C-sweat (+) ivacaftor. We assume that glands sometimes secrete amounts of sweat that fail to exceed the threshold resulting from subtractive processes [10]. To assess this assumption, we dichotomized the C-sweat response as 0, if there is no observed secretion, and 1, otherwise. With this recoding, the average recoded C-sweat response is simply the *probability* that a gland produces C-sweat on a test, and we use this probability as an alternative index of C-sweat production (see **Appendix S1**).

Note that this method is useful for testing statistical significance when many glands do not respond, the situation with CF subjects (−) ivacaftor, but becomes insensitive as the response rate increases and P(C-sweat) approaches 1. For example, WT and HZ both have P(C-sweat) ≈ 1, but C/M ratios of 1 and 0.5 respectively. The upper limit for the utility of this method is a function of CFTR functional levels and gland secretory capacity (M-sweat rate) and remains to be determined.

## Results

For six CF subjects each carrying one G551D mutation, CFTR-independent M-sweating was imaged for 15 minutes, allowing us to identify 58–143 sweat glands per subject and measure the M-sweat rates for each gland. The same glands were then stimulated with the β-adrenergic cocktail to stimulate CFTR-dependent C-sweating, which was imaged for 30 min. Two subjects were tested (+/−) ivacaftor, two only (+) ivacaftor and two only (−) ivacaftor (**Table 1**). Images of M-sweat and C-sweat bubbles are shown for each G551D CF subject in **Fig. 1**.

**Table 1:** G551D/F508del subjects.

			Gland numbers			Gland volumes/rates				Ratios			
ID	G	TestDate	MCh glands (n)	Cktl glands (n)	C/M n glands (%)	MCh Avg. Final Vol. (nl/gland)	MCh Rate Estimate nl/gl/min	Cktl Avg Final Vol (pl/gl)	Cktl Rate (pl/gl/min)	C mean/M mean	% WT	% WT (Highest 12 M-sweat)	% WT (Hi-12, Loss-Corrected)
**S1 G551D/F508del (−) ivacaftor**													
S1	M	10/20/2011	71	0	0%	75.83	5.06	0	0	0.00%	0.00%	-	—
S1	M	1/13/2012	85	0	0%	64.58	4.31	0	0	0.00%	0.00%	-	—
S1	M	2/4/2012	75	0	0%	71.34	4.76	0	0	0.00%	0.00%	-	—
**S1 (−) Averages**			**77**	**0**	**0%**	**70.58**	**4.71**	**0**	**0**	**0.00%**	**0.00%**	**0.00%**	**0.00%**
**S1 G551D/F508del (+) ivacaftor**													
S1	M	3/23/2012	82	13	16%	70.99	4.73	152	5.06	0.11%	0.42%	-	—
S1	M	4/10/2012	71	15	21%	91.91	6.13	105	3.52	0.06%	0.22%	-	—
S1	M	4/17/2012	66	11	17%	97.02	6.47	110	3.66	0.06%	0.22%	-	—
**S1 (+) A xverages**			**73**	**13**	**18%**	**86.64**	**5.78**	**122**	**4.08**	**0.07%**	**0.29%**	**0.64%**	**1.59%**
**S2 G551D/F508del (−) ivacaftor**													
S2	F	2/2/2012	99	0	0%	28.61	1.91	0	0	0.00%	0.00%	-	—
S2	F	3/20/2012	100	0	0%	29.16	1.94	0	0	0.00%	0.00%	-	—
**S2 (−) Averages**			**99.5**	**0**	**0%**	**28.88**	**1.93**	**0**	**0**	**0.00%**	**0.00%**	**0.00%**	**0.00%**
**S2 G551D/F508del (+) ivacaftor**													
S2	F	5/8/2012	82	4	5%	35.55	2.37	11	0.37	0.02%	0.06%	-	—
S2	F	5/15/2012	90	1	1%	29.16	1.94	2	0.07	0.00%	0.01%	-	—
**S2 (+) Averages**			**86**	**2.5**	**3%**	**32.36**	**2.16**	**7**	0.22	**0.01%**	**0.04%**	**0.13%**	**2.75%**
**S3 G551D/F508del (+) ivacaftor**													
**S3**	**F**	**7/24/2012**	**90**	**21**	**23%**	**45.03**	**3.00**	**95**	**3.18**	**0.11%**	**0.41%**	**0.58%**	**2.27%**
**S5 G551D/F508del (+) ivacaftor data**													
**S5**	**M**	**11/2/2012**	**58**	**29**	**50%**	**78.94**	**5.26**	**703**	**23.42**	**0.45%**	**1.74%**	**3.07%**	**4.11%**
**S6 G551D/F508del (−) ivacaftor data**													
**S6**	**F**	**2/2/2012**	**143**	**0**	**0%**	**14.37**	**0.96**	**0**	**0**	**0.00%**	**0.00%**	**0.00%**	**0.00%**
**S7 G551D/F508del (−) ivacaftor data**													
**S7**	**F**	**2/13/2012**	**123**	**0**	**0%**	**32.16**	**2.14**	**0**	**0**	**0.00%**	**0.00%**	**0.00%**	**0.00%**
**Summary (−) ivacaftor: 4 G551D subjects**													
**Sums or Averages**			**852**	**0**	**0%**	**36.50**	**2.43**	**0**	**0**	**0.00%**	**0.00%**	**0.00%**	**0.00%**
**Summary (+) ivacaftor 4 G551D subjects**													
**Sums or Averages**			**906**	**317**	**29%**	**42.93**	**2.84**	**267**	**6.80**	**0.29%**	**1.15%**	**1.84%**	**4.07%**

Summary of results for CF subjects with G551D mutations. Six adult G551D CF subjects were tested. Table is arranged by subject and condition, each row shows the results for a single test or means/sums when multiple tests were performed. Subjects had been taking ivacaftor for at least 3 weeks prior to (+) ivacaftor testing. Columns labeled ‘gland numbers’ show the number of glands that secreted to an intradermal injection of MCh, then to the β-adrenergic cocktail at the same site (C-sweat), and the ratio of the two expressed as a percentage. Columns labeled ‘gland volumes/rates’ show the average final M-sweat or C-sweat volumes per gland expressed as nanoliters for a 15 min period (M-sweat) or 30 min period (C-sweat) followed by average rates (per min, per gland). The 4 columns labeled ‘ratios’ show: 1) the mean C-sweat rate as a percent of the mean M-sweat rate; 2) the C/M ratios as a percentage of the average C/M ratio of 0.2651 obtained for 6 control subjects [10]; 3) that value for the 12 glands having the highest M-sweat rates; and 4) the value for the 12 highest glands after correcting for sweat volume loss.

### G551D subjects: within-subject comparisons (−) and (+) ivacaftor

**Figure 2:**
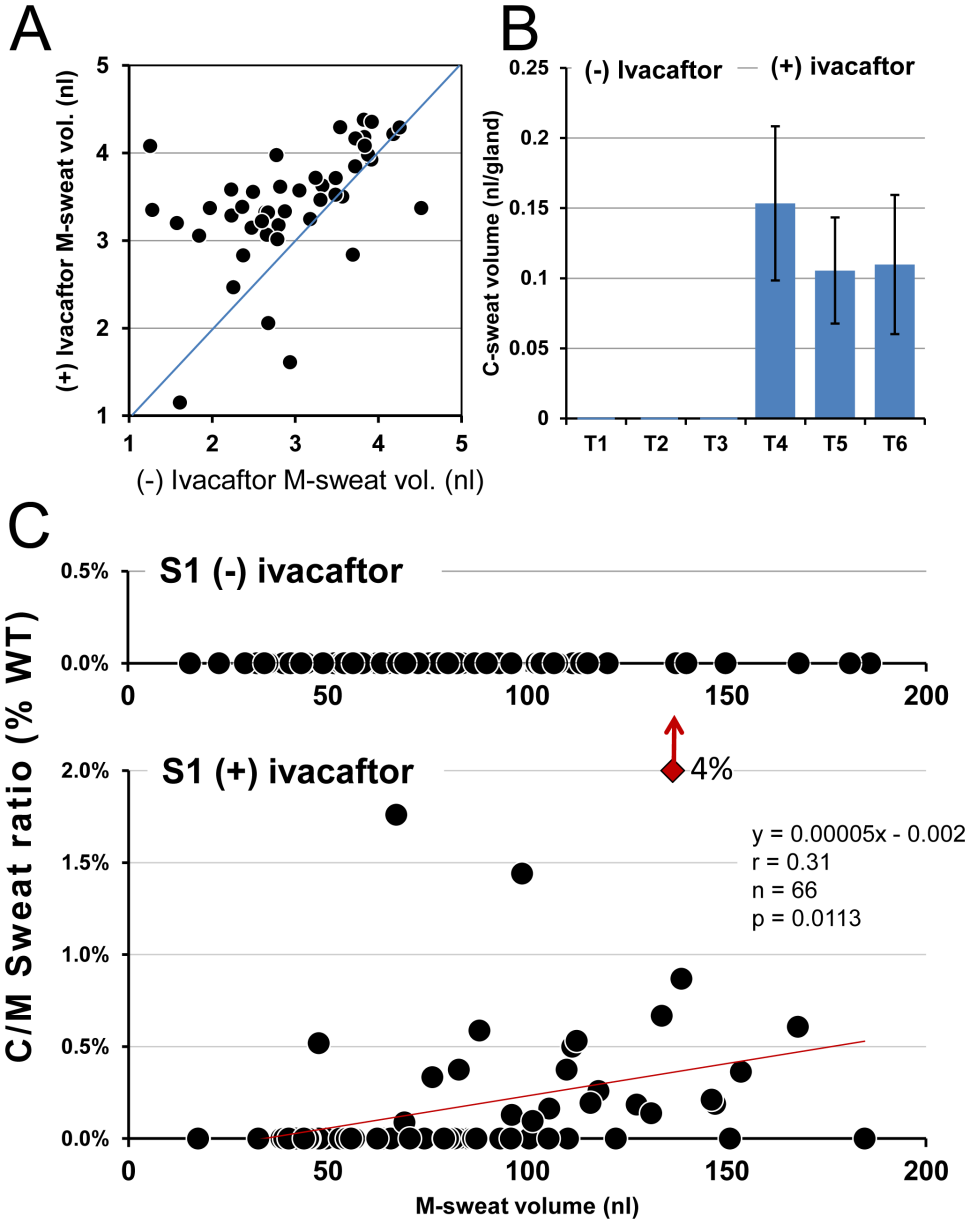
M- and C-sweat secretion (±) ivacaftor for CF subject 1: male, F508del/G551D. (A) Larger M-sweat responses (+) ivacaftor were seen (uniquely) in this subject. Scatter plot shows gland-by-gland volumes measured 7.5 min post MCh injection before any appreciable mergers for 46 glands. Each point is the average of 2–3 measures (2.93 measures per gland per condition), log transformed. Value (−) ivacaftor are on the x-axis and (+) ivacaftor on the y axis. Mean volume at the 7.5 min time point was 28.7±20 vs. 38.35±20 nl^−gland^, *p* = 0.004, paired t-test on log transformed data. Diagonal line shows equivalence. (B) Bar graphs showing mean C-sweat volumes ± ivacaftor; 3 tests in each condition; zero C-sweat seen (−) ivacaftor. (C) Gland-by-gland sweat secretion (±) ivacaftor. Each point in these graphs is jointly determined by the mean of M-sweat responses (x axis) and C-sweat/M-sweat ratios expressed as a% of the mean WT C/M ratio (y axis). Top graph with truncated y axis shows measurements (−) ivacaftor. Each point is mean ratio measured in 1–3 tests (mean 2.1 tests) for each of 94 identified glands. C-sweating and hence ratio was zero for all glands. Bottom graph, (+) ivacaftor. 25/66 glands produced C-sweat on 1–3 trials (average 1.56 trials). The dark outlined red diamond and arrow show an outlier gland (G19) which secreted ∼10 times more than the mean of all responding glands on each of 3 (+) ivacaftor trials. Red line is linear fit.

**Subject 1** (**S1**, male, F508del/G551D) was tested 3 times in each condition. (This is the preferred paradigm for testing, but it could not be used with all subjects because of limited subject availability.) M-sweat is considered to be CFTR-independent [16], [17], but unexpectedly, S1's M-sweat rates were significantly larger (+) ivacaftor (78.0 versus 58.5 nl^−gland^, p<.01; see **Fig. 2A**). Glands differed significantly in their typical M-sweat rates (p<.0001), but not in their sensitivity to ivacaftor (p>.38). Indeed, M-sweating, presumably reflecting gland size, varied ∼10 fold, as previously observed [10]. There was also significant variation in average M-sweat rates across weeks (p<.05); but in this subject the 6 tests were spread out across a 6 month period.

Ivacaftor caused a qualitative difference in C-sweating. No gland produced C-sweat in any of 3 tests (−) ivacaftor, but (+) ivacaftor 16–21% of the glands responded (**Table 1**) and produced an average of 0.1–0.15 nl^−gland^ on each of 3 tests (**Fig. 2B**). (Glands with zero C-sweat were included in the averages). As noted in **Appendix S1**, we dichotomized the C-sweat response, and then replaced by ‘1’ one of the ‘0’s (i.e., non-secretions) in the (−) ivacaftor condition. A logistic mixed model analysis of the minimally perturbed responses showed the effect of ivacaftor was significant [P(C-sweat) was estimated to be.1035 and.0009 in the (+) and (−) ivacaftor conditions, respectively, p<.0005]. Here too, glands (p<.0001), but not weeks (p>.99), differed significantly in the probability of a C-sweat response.

**Fig. 2C** presents data for S1 on a gland-by-gland basis, in which each point represents the joint mean values for C-sweat and M-sweat for a single gland. The top, truncated graph of **Fig. 2C** shows that no glands produced C-sweat in the absence of ivacaftor across the full range of M-sweat responses (0/231 = 0% response rate based on a mean of 2.35 measurements for each of 98 glands), hence all points are arrayed along the x axis according to their M-sweat responses. The bottom graph of **Fig. 2C** shows the results (+) ivacaftor. C-sweat responses were observed for 24 glands on at least one test with an overall response rate of 17.9% and C/M ratios 0%–4% of the average WT ratio. **Fig. 2C** also shows that the C/M ratio was not constant across the glands, but increased significantly as a function of the M-sweat rate (r = 0.31, p<0.02), see also [10]. The positive relationship between C/M and M, which was significant for all but one subject, was used to arrive at better estimates of the% of CFTR function restored by ivacaftor (see discussion).

As an additional means to estimate the level and significance of CFTR function that is restored by ivacaftor, we developed a prediction equation based on the gland's M-sweat response (+) ivacaftor, as discussed in **Appendix S1**. If the significant differences in P(C-sweat) across glands is due mainly to factors, e.g., gland size, that are reflected in the M-sweat response (+) ivacaftor, then one would expect that, when M-sweat response is included as a predictor, ‘gland’ would no longer be significant as a random effect. Similarly, to the extent that differences among weeks are reflected in the M-sweat response, ‘week’ would no longer be significant as a random effect. A logistic mixed model analysis showed that M-sweat response (on a log scale) was a significant predictor of P(C-sweat) (b = 2.43, p<.0001) in the (+) ivacaftor condition. Further, neither ‘gland’ (p>.17) nor ‘week’ (p≈1) was significant, suggesting that differences in CFTR function (i.e., in the CFTR-dependent C-sweat response) across glands and weeks can be adequately accounted for, or ‘explained’, by differences in the CFTR-independent M-sweat responses of the glands. On the log scale, M-sweat levels vary from 0 to 5.527, and the corresponding probabilities of a C-sweat response are.00006 and.23

**Figure 3:**
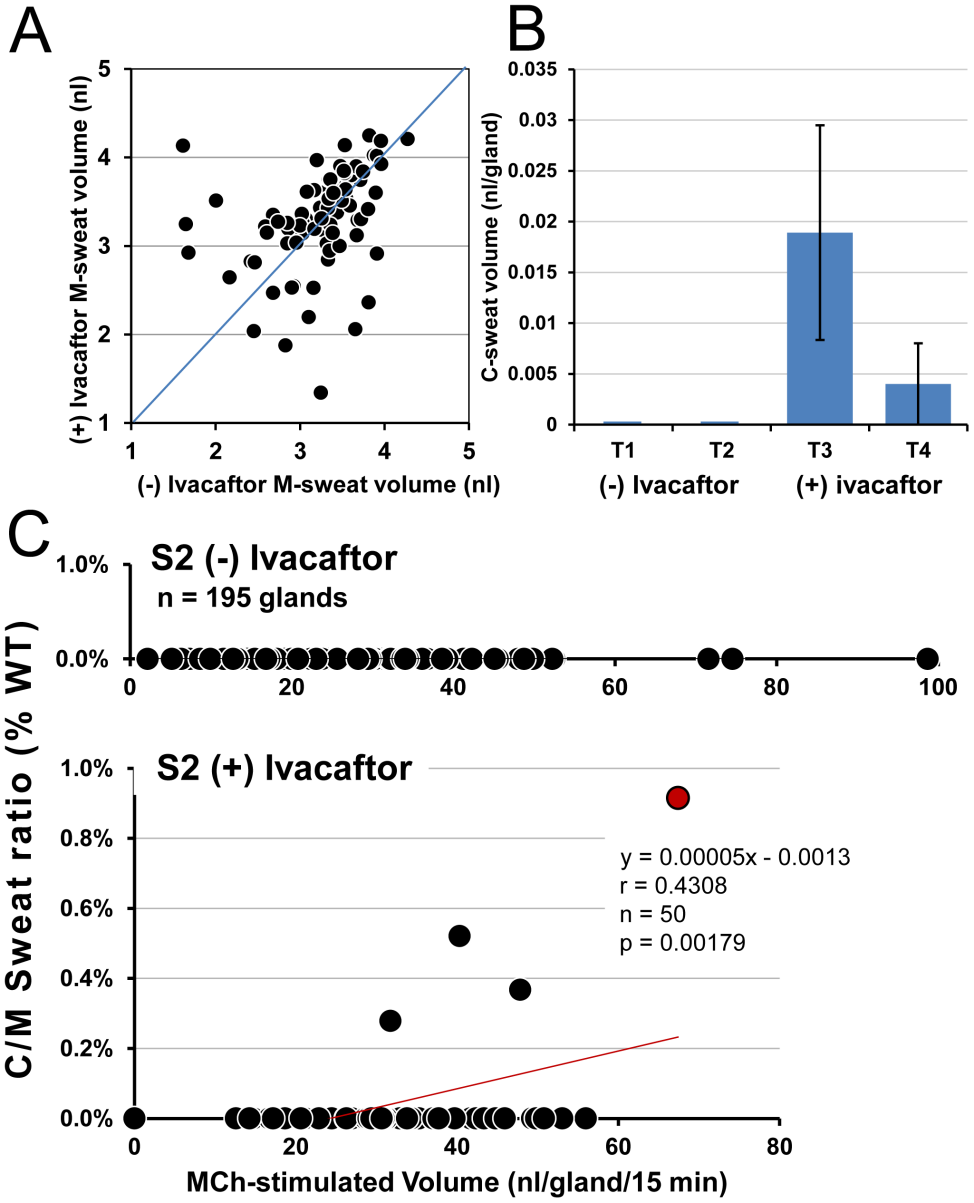
M- and C-sweat secretion (±) ivacaftor for CF subject 2: female, F508del/G551D. (A) M-sweat responses were unchanged by ivacaftor in this subject. Each point is log transformed values for a single pre-test and mean of 2 post-test measures for each of 87 glands. Mean volumes were 28.2±12.5 vs. 31.9±14.6 nl^−gland^, pre and post, n = 87 glands *p* = 0.12, paired t-test on log transformed data, plotted as in Fig. 2A. (B) Bar graphs show C-sweat volumes (mean ± SEM) for (±) ivacaftor conditions; 2 tests in each condition. One (−) ivacaftor test was at a different site. (The means are based on glands 1–50). (C) Gland-by-gland C-sweat secretion ± ivacaftor. Each symbol shows correlations for each gland between mean M-sweat volumes (x axis) vs. C-sweat/M-sweat ratio expressed as a% of mean WT value (y axis). (Top graph, truncated y axis) shows measurements (−) ivacaftor. Each point is M-sweat volume for a single test for 195 identified glands at two sites; C-sweat was zero. (Bottom graph) shows measurements (+) ivacaftor (mean of 2 tests). Four of 87 glands produced C-sweat, only one gland secreted on both tests (red symbol). Red line is linear fit. This was the smallest response observed for any subject (+) ivacaftor.

**Subject S2** (**S2**, female, F508del/G551D) was tested twice in each condition. Unlike S1, S2's M-sweat values did not differ between (+) and (−) ivacaftor (26.5 versus 25.3 nl^−gland^, p>.9; see **Fig. 3A**). C-sweating for S2 was evaluated in the same way as for S1. **Fig. 3B** shows that no C-sweat volume was produced (−) ivacaftor, and only a tiny average volume (<20 pl^−gland^) was produced (+) ivacaftor because only 1–4 out of 90 glands responded (**Fig. 3C, D**). A logistic mixed model analysis of the minimally perturbed responses showed a non-significant effect of ivacaftor (p>.13). Here too glands (p<.0001), but not weeks (p>.99), differed significantly in the probability of a C-sweat response. In predicting P(C-sweat) from M-sweat response (+) ivacaftor, a logistic mixed model analysis yielded unreliable estimates of the variances of the random effects for gland and week, most likely because less than 3% of glands yielded observable levels of C-sweat. A simple logistic regression without random effects did show that M-sweat response (on a log scale) was a significant predictor of P(C-sweat) (b = 3.88, p<.05) (see discussion).

### Two additional G551D subjects tested only (+) ivacaftor

**Figure 4:**
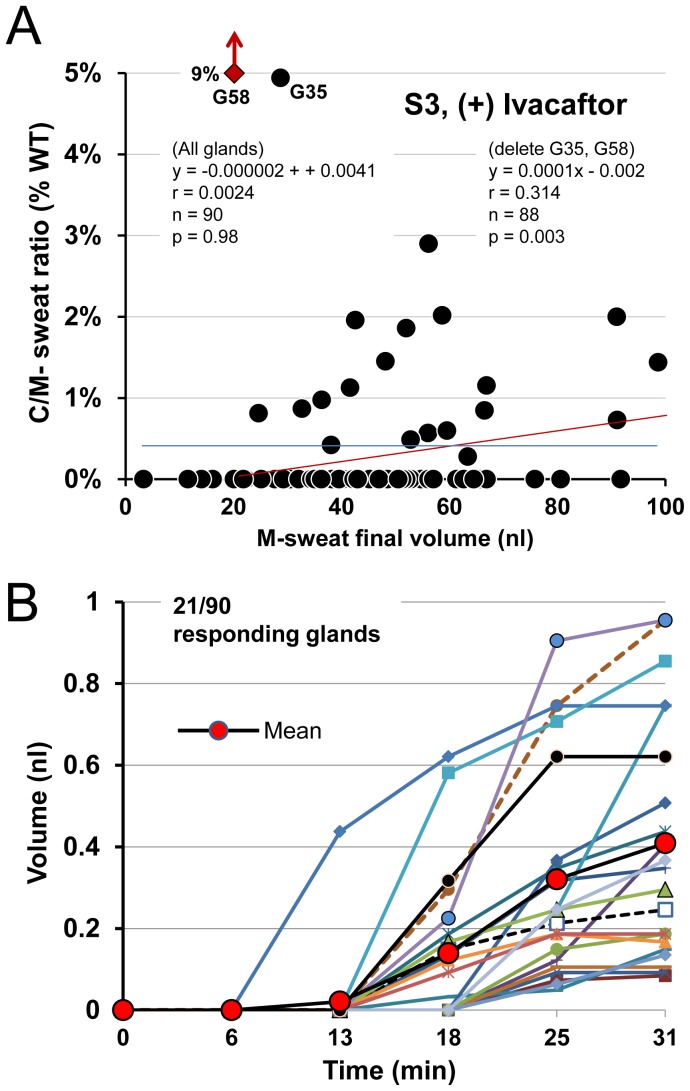
C-sweat secretion (+) ivacaftor in CF subject 3: female, F508del/G551D. S4 was tested only once, (+) ivacaftor. (A) Gland-by-gland correlations of M- and C-sweating using the same conventions as above. M-sweat was measured for 90 glands of which 21 also produced C-sweat with C/M ratios of 0.3% to 5% of the mean WT ratio. (B) Volume vs. time plots for C-sweating of each responding gland. β-adrenergic cocktail injected at time 0, and no observations were made during the first 3 min when atropine was taking effect and when the site was rinsed and dried before reapplying oil. Note slow time course of response.

Subject 3 (**S3**, female, F508del/G551D) was tested only once. M-sweating was measured for 90 glands, of which 21 (23%) produced measureable C-sweat with C/M ratios 0.3%–9% of the mean WT ratio **(** **Fig. 4A**). The average C/M for all glands was 0.41% WT. **Fig. 4B** plots volumes vs. time for C-sweat from each of the 21 responding glands. Most responding glands had long post-injection latencies, and many continued to increase throughout the 30 min period. This subject (or test) was unusual in that two glands with low M-sweat rates had high C-sweat rates, leading to unusually high C/M ratios and resulting in a non-significant association between C/M ratio and M-sweat rate (r = 0.002, p = 0.98). Excluding those two glands gave r = 0.314, p = 0.003).

**Figure 5:**
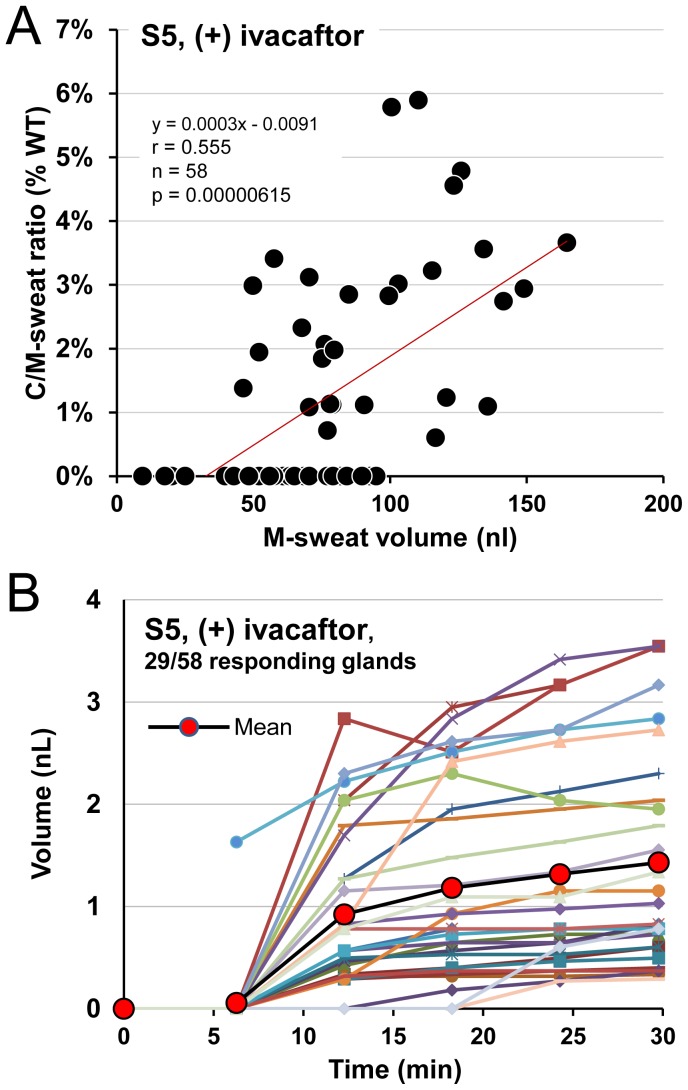
C-sweat secretion (+) ivacaftor in CF subject 5: male, F508del/G551D. S5 was tested only once, (+) ivacaftor. (A) Gland-by-gland correlations of M- and C-sweating using the same conventions as previously. M-sweating was measured in 58 glands. Of these, 29 (50%) also produced C-sweat, with C/M ratios of 0.6% to 5.9% of the mean WT ratio. (B) Volume vs. time plots for β-stimulated, C-sweating. β-adrenergic cocktail injected at time 0.

Subject 5 (**S5**, male, F508del/G551D), was tested only once (+) ivacaftor. M-sweating was measured in 58 glands, of which 29 (50%) produced C-sweat, giving C/M ratios of 0.6% to 5.9% of the mean WT ratio (**Fig. 5A**). The time course of increases in C-sweat bubble volumes in this subject was similar to S4 (**Fig. 5B**). (Time courses of responding glands from S1 and S2 were similar to S3 and S5, data not shown). The C/M ratio increased significantly as a function of the M-sweat rate (r = 0.555, p = 0.000006).

In predicting P(C-sweat) from M-sweat response (+) ivacaftor, a logistic mixed model analysis showed that M-sweat response (on a log scale) was a significant predictor of P(C-sweat) (S3: b = 1.43, p<.05; S5: b = 3.73, p<.01).

### Two additional G551D subjects tested only (−) ivacaftor

**Figure 6:**
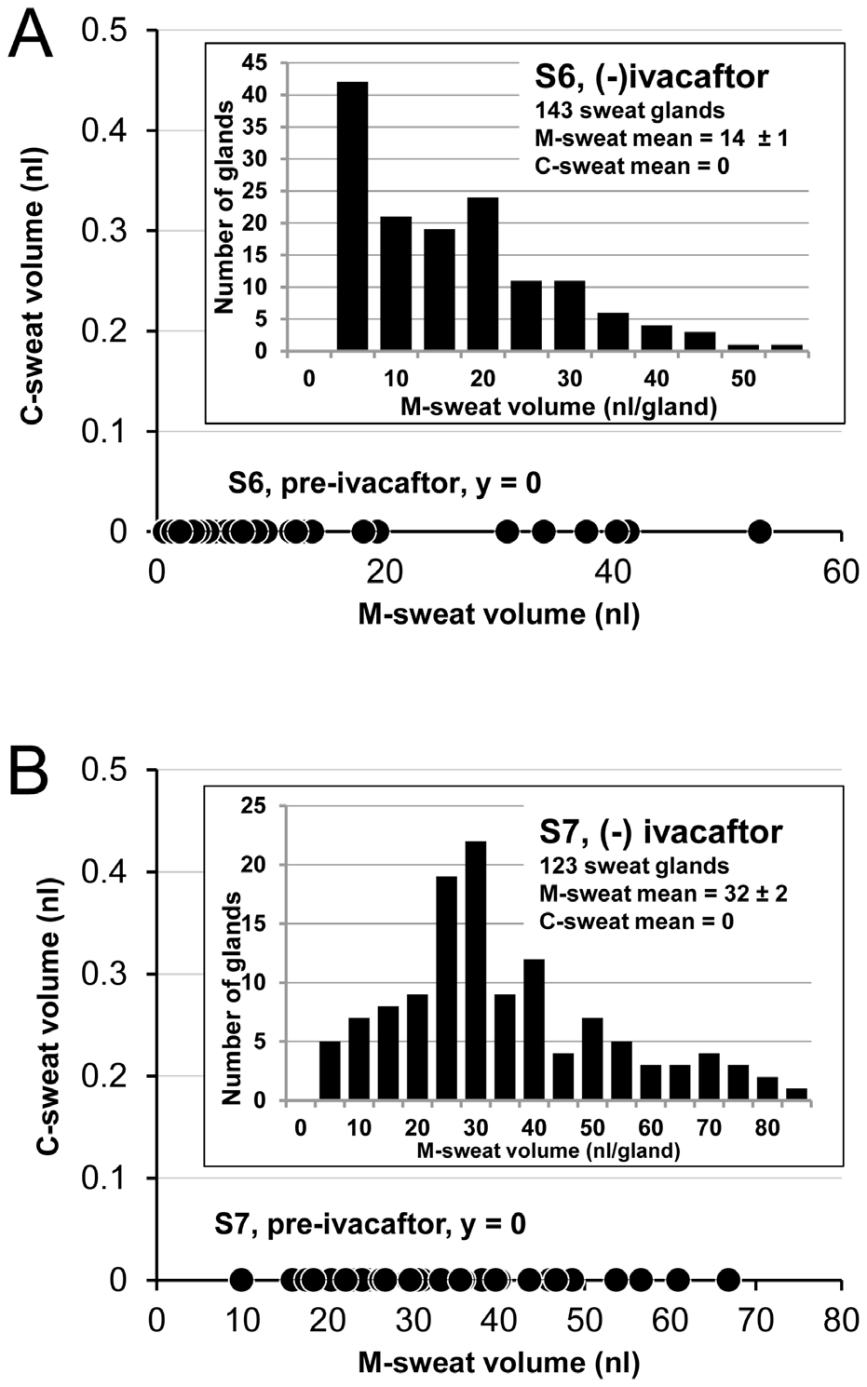
Absence of C-sweat secretion (−) ivacaftor in two G551D CF subjects. (A) S6: female, F508del/G551D. Each symbol in the main graph shows M-sweat volumes produced by a single sweat gland (x-axis) and C-sweat (truncated y axis, all C-sweat values were zero). The distribution of M-sweat volumes is shown in the inset. (B) S7: female, F508del/G551D. Same format as for S6.

Two female G551D CF subjects, **S6** and **S7**, were each tested once only (−) ivacaftor. They did not return for repeat pre-testing or since starting ivacaftor. Each subject had abundant sweat glands, none of which responded to the β-adrenergic cocktail with any measureable C-sweat (0/266 glands, main graphs of **Fig. 6A, B**). The insets show the distribution of M-sweat volumes for these two subjects; note the prevalence of low-secreting glands for S6.

### Within-subject study of an R117H-5T subject (−) and (+) ivacaftor

**Figure 7:**
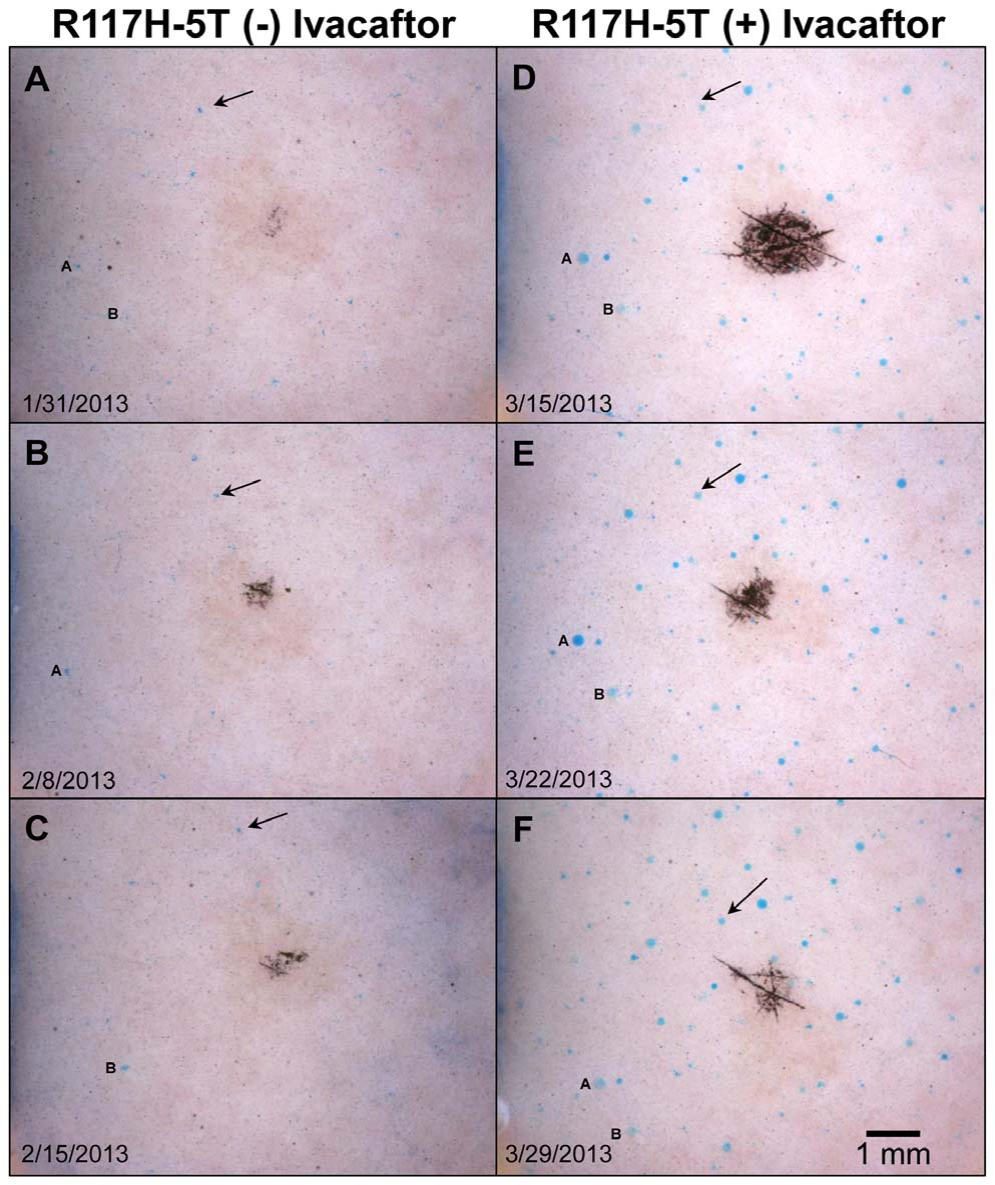
Images of C-sweat responses for CF subject 8: male, I507del/R117H-5T (±) ivacaftor (N of 1 trial). S8 was tested 3 times (−) ivacaftor and 5 times (+) ivacaftor. Only the first 3 (+) ivacaftor tests are shown. (A-C) (−) ivacaftor; (D-F) (+) ivacaftor. Each image shows the field 30 min after the cocktail injection. A light freckle served as the landmark, with an ink spot on the freckle to improve registration and focusing. For image (F) the ink spot was too high causing the field to shift down. In this pancreatic sufficient subject, a few glands marked as A, B, or arrows) produced small amounts of C-sweat (−) ivacaftor.

Subject 8 has genotype I507del/R117H-5T. The I507del mutation does not traffic [18], [19] and so is predicted to be unresponsive to ivacaftor, allowing us to assess the *in vivo* effect of ivacaftor on the R117H-5T mutation in isolation. S8 was tested 3 times (−) ivacaftor and 5 times (+) ivacaftor **(** **Table 2** **)**. In the (−) ivacaftor condition, the C-sweat response to the β-adrenergic cocktail was not completely absent for this pancreatic sufficient subject. One gland (G43, arrows in **Fig. 7**) in the region of interest (ROI) and two glands outside the ROI (glands A, B in **Fig. 7A-C**) responded to cocktail in the (−) ivacaftor condition on all 3 tests. Using just the glands in the ROI gave a response rate of 3/153 (1.9%).

**Table 2:** Summary of results for R117H-5T CF subjects and the healthy control.

			Gland numbers			Gland volumes/rates				Ratios			
ID	G	Test Date	MCh glands (n)	Cktl glands(n)	C/M n glands (%)	MCh Avg. Final Vol. (nl/gland)	MCh Rate Estimate nl/gl/min	Cktl Avg Final Vol (pl/gl)	Cktl Rate (pl/gl/min)	C mean/M mean	% WT	% WT (Highest 12 M-sweat)	% WT (Hi-12, Loss-Corrected)
**S4 G551D/R117H-5T (+) ivacaftor data**													
S4	F	8/22/2012	37	8	22%	16.05	1.07	100	3.33	0.31%	1.22%	-	—
S4	F	11/6/2012	32	6	19%	10.66	0.69	30	1.00	0.14%	0.55%	-	—
S4	F	4/30/2013	37	11	30%	13.57	0.57	150	5.00	0.55%	2.16%	-	—
**S4 (+) Averages**			**35.33**	**8.33**	**23%**	**13.43**	**0.78**	**93**	**3.11**	**0.35%**	**1.31%**	**2.13%**	**7.83%**
**S8 R117H/I508del (−) ivacaftor**													
S8	M	1/31/2013	52	1	2%	46.76	3.12	6	0.19	0.01%	0.02%	—	—
S8	M	2/8/2013	54	1	2%	49.88	3.33	12	0.39	0.01%	0.04%	—	—
S8	M	2/15/2013	50	1	2%	59.51	3.97	4	0.14	0.00%	0.01%	—	—
**S8 (−) Averages**			**52**	**1**	**0%**	**52**	**3**	**7**	**0.24**	**0.01%**	**0.03%**	**0.06%**	**1.50%**
**S8 R117H/I508del (+) ivacaftor**													
S8	M	3/15/2013	53	28	53%	41.40	2.76	531	17.70	0.64%	2.42%	—	—
S8	M	3/22/2013	53	45	84%	54.35	3.62	766	25.55	0.71%	2.66%	—	—
S8	M	3/29/2013	49	42	86%	53.88	3.59	1146	38.20	1.06%	4.01%	—	—
S8	M	6/17/2013	53	43	81%	57.84	3.86	645	21.49	0.56%	2.10%	—	—
S8	M	6/24/2013	53	40	75%	53.85	3.59	474	15.80	0.44%	1.66%	—	—
**S8 (+) Averages**			**52.2**	**39.6**	**76%**	**52.26**	**3.48**	**712**	**23.75**	**0.68%**	**2.57%**	**4.51%**	**5.89%**
**Healthy Control**													
WT	F	3/14/2013	51	50	98%	35.94	2.40	9687	323	13%	51%	—	—
WT	F	3/25/2013	51	51	100%	36.98	2.47	9371	312	13%	48%	—	—
WT	F	3/28/2013	51	51	100%	28.81	1.92	11519	384	20%	75%	—	—
WT	F	5/2/2013	51	50	98%	36.48	2.43	10540	351	14%	54%	—	—
**WT Averages**			**51**	**50.5**	**99%**	**34.55**	**2.30**	**10279**	**343**	**15%**	**57%**	**60.65%**	**62.97%**

Table is arranged using same format as Table 1 and shows data for S8, S4 and the healthy control.

**Figure 8:**
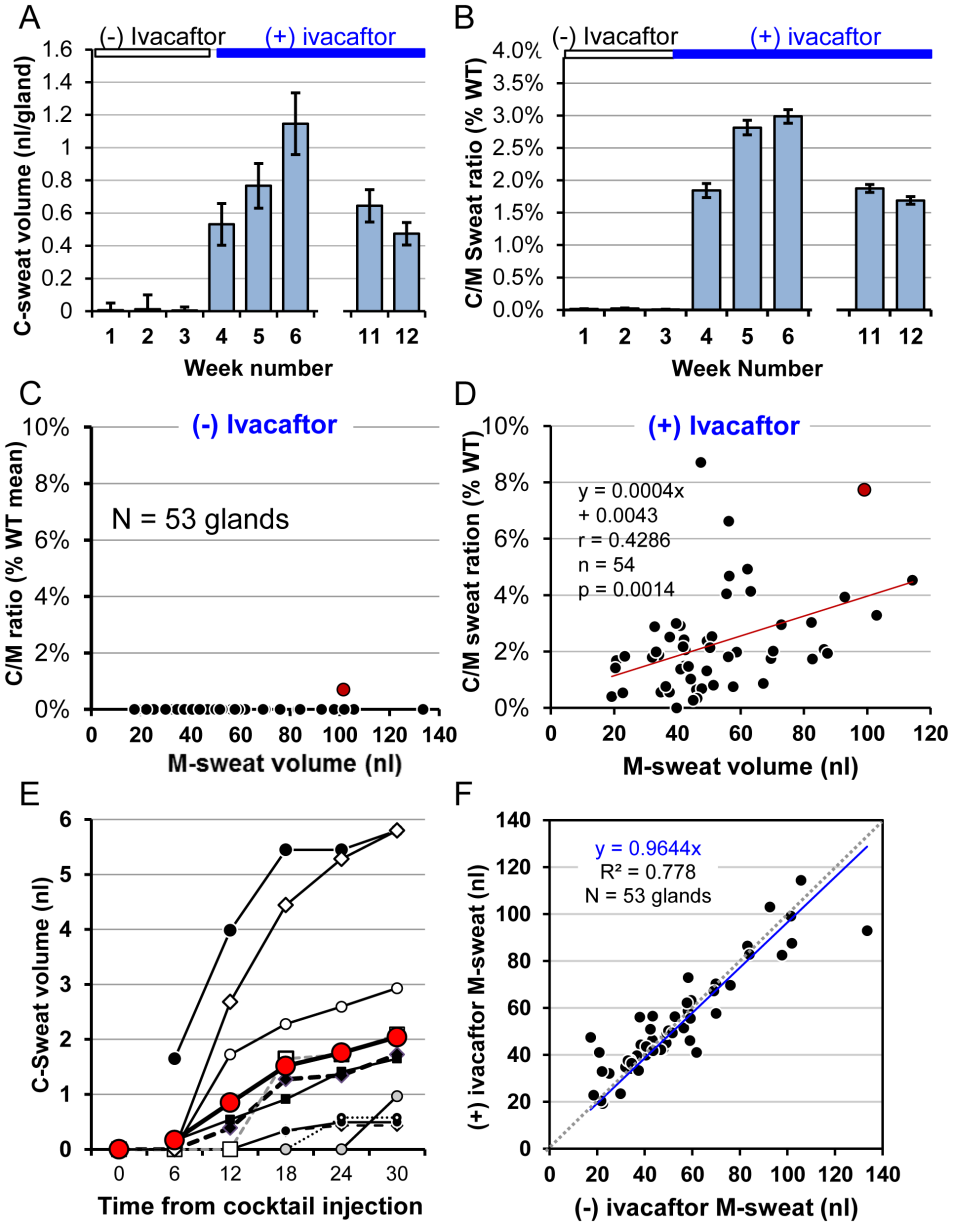
Summary data for S8, R117H-5T, (±) ivacaftor. (**A**) Mean ± SEM for the final (30 min) volume of C-sweat secretion per gland (50–53 glands measured for each test) (±) ivacaftor. (B) C-sweat/M-sweat ratios expressed as percent of the WT mean. Results are means ± SEM for 49–53 glands per test. (C-D) Gland-by-gland correlations of M-sweat and C-sweat responses in the absence (C), and presence (D) of ivacaftor. Each symbol represents a single gland and shows its average C/M ratio expressed as a% of the WT average (y-axis) vs. its mean M-sweat volume (x-axis). A single gland (G43) in the original ROI produced C-sweat (−) ivacaftor (red symbol); this gland had the second highest C/M ratio in the (+) ivacaftor condition (red symbol). In the (+) ivacaftor condition, 92% of the glands in the ROI produced C-sweat on at least one of the 3 trials, at C/M ratios 0.3%–8.7% of the mean WT C/M ratio. (E) C-sweat volume vs. time for 10 responding glands selected to represent the range of responses for S8 (+) ivacaftor. β-adrenergic cocktail injected at time 0. (F) Absence of ivacaftor effect on M-sweating in this subject. Each point represents a single identified sweat gland, and shows the average M-sweat volumes from 3 tests (−) ivacaftor (x-axis) and 5 tests (+) ivacaftor (y-axis). Dashed 45 degree line indicates equivalence; linear fit does not differ significantly from it.

**Figure 9:**
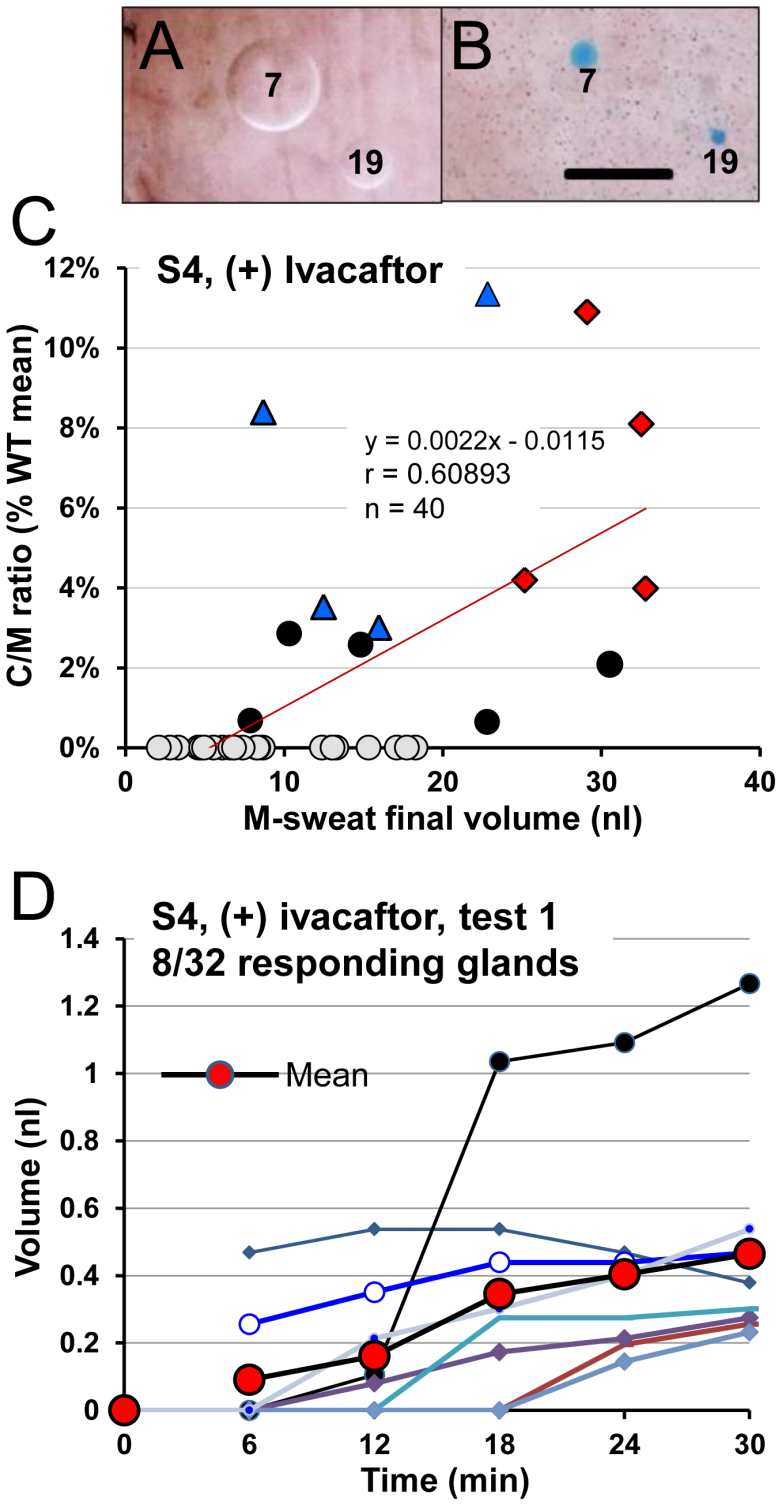
Summary data for S4, a female CF subject having two potentially responsive mutations: G551D & R117H-5T who was tested 3 times (+) ivacaftor. (A) Examples of M-sweat. (B) Examples of C-sweat. Calibration  = 0.5 mm. (C) Gland-by-gland correlations of M- and C-sweating using same conventions as previous figures. Symbols: Filled circles, C-sweat on 1/3 trials, blue triangles, 2/3 trials and red diamonds, 3/3 trials. (D) Volume vs. time plots for C-sweating from a single experiment (test 1). β-adrenergic cocktail injected at time 0.

C-sweating increased unequivocally in the (+) ivacaftor condition (**Figs. 7** **,** **8**). After starting ivacaftor, 53–85% of the glands responded on each test, with an average response rate of 75±15% (SD) across the 5 tests. **Fig. 8A** shows the mean final volumes of C-sweat for each of 8 tests. C-sweat volume (−) ivacaftor could be measured in only one gland so the average was almost undetectable (0.007 nl^−gland^ accumulated over the 30 min observation period), but in the presence of ivacaftor 53 glands produced an average of 0.71±0.04 nl^−gland^ during the 30 min period. The C/M-sweat ratio expressed as a% of the WT mean was 0.01% (−) ivacaftor and was 2.2±0.6% (SD) (+) ivacaftor (**Fig. 9B**). Mean responses for each of the 53 glands ( ± ) ivacaftor are shown in **Fig. 9C, D** where each point is the mean of 5 M-sweat responses (x axis) and 5 C/M ratios as a% of the mean WT value (y axis). The response rate for 10 selected glands is shown in **Fig. 8E**.

As we did for S1 and S2, we performed an additional analysis by dichotomizing the C-sweat response before conducting a logistic mixed model analysis of the raw responses. The effect of ivacaftor was significant [P(C-sweat) was estimated to be.8065 and.0085 in the (+) and (−) ivacaftor conditions, respectively, p<.0001]. Like S2 but unlike S1, S8's M-sweat values did not differ between (+) and (−) ivacaftor (46.1 versus 46.0 nl^−gland^, p>.9; see **Fig. 8F**). Consistent with the other subjects tested in this way, glands differed significantly from one another in their typical M-sweat rates (p<.0001), but not in their sensitivity to ivacaftor (p>.45). In predicting P(C-sweat) from M-sweat response (+) ivacaftor, M-sweat (on a log scale) was a significant predictor of P(C-sweat) (b = 2.00, p<.0001), with M-sweat levels varying from −0.064 to 4.998, and P(C-sweat) levels from 0.0013 to 0.9711 (**Appendix S1**).

### Study of a CF subject with two responsive mutations: G551D/R117H-5T

Subject 4 (**S4**, female, G551D/R117H-5T) has two mutations that are each expected to respond to ivacaftor. This led us to expect a greater response, but she also had the fewest glands and lowest M-sweat rate of any subject, which predicted a smaller C-sweat response as noted above. S4 was tested 3 times (+) ivacaftor (**Table 2**). We identified 40 glands; 2 are shown in (**Fig. 9A, B**). M-sweating was measured from 32–37 of these gland on each trial (the lowest number for any subject). Of these, 13 (40%) also produced C-sweat on at least one of the three tests. On average, 22% of glands (range 15–30%) in a given trial produced C-sweat. The C/M ratios for responding glands ranged from 0.7% to 11.4% of the mean C/M ratio for WT subjects (**Fig. 9C**); the average ratio for all glands was 1.31% WT. The response time course (**Fig. 9D**) was similar to other subjects, presumably reflecting the offsetting factors of a larger response to ivacaftor and a smaller M-sweat rate. The C/M ratio increased significantly as a function of the M-sweat rate (r = 0.61, p = 0.00003). M-sweat response (on a log scale) was a significant predictor of P(C-sweat) (b = 2.03, p<.0001), with M-sweat levels varying from −0.60 to 3.78, and the P(C-sweat) levels from 0.0005 to 0.7876 (**Appendix S1**).

### Responses of a control subject

**Figure 10:**
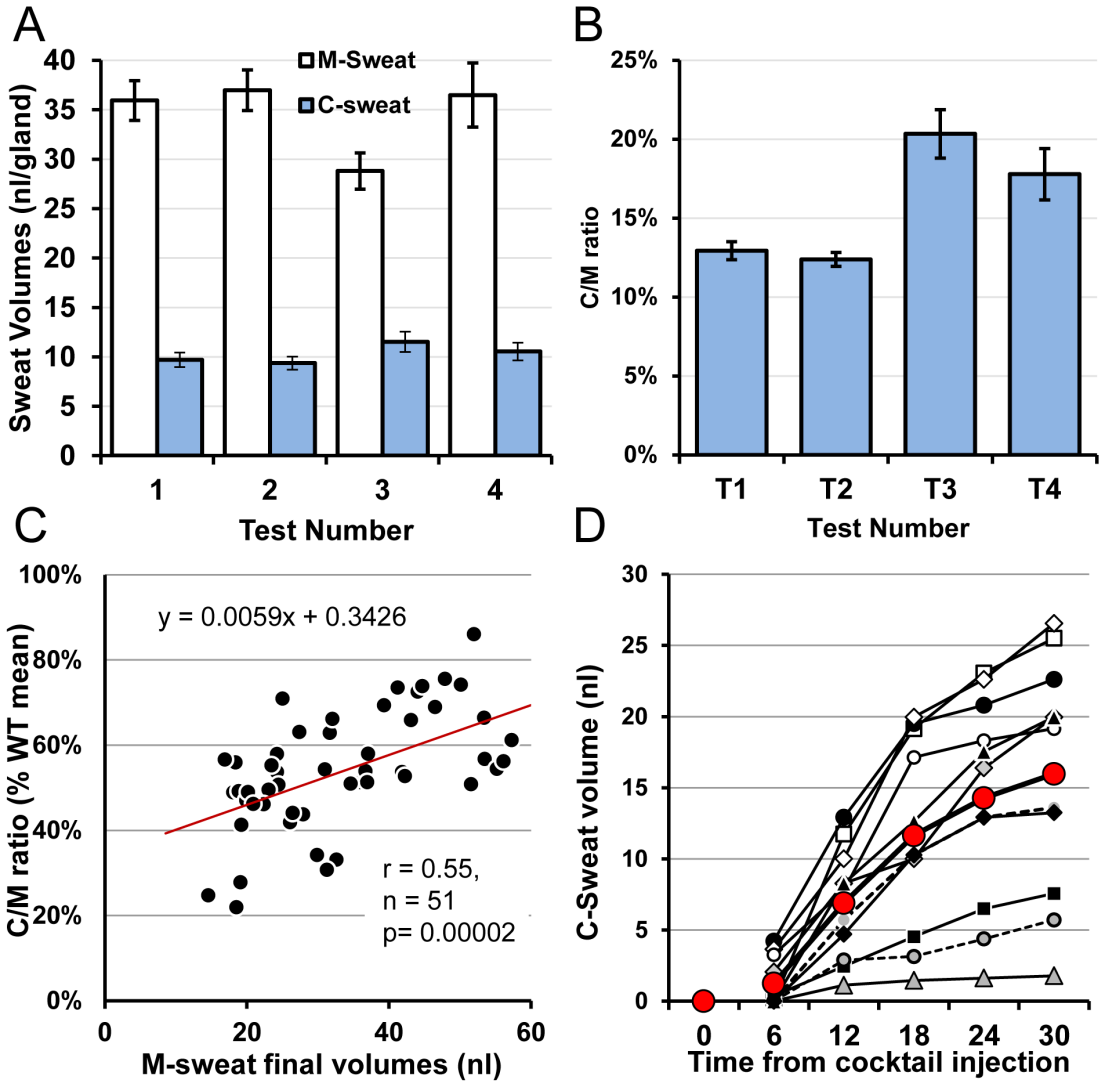
M- and C-sweating in a female, healthy control subject. (A) M-sweat and C-sweat responses for each of 4 tests (Mean ± SEM, n = 51 glands). Each open bar is the average final M-sweat volume in response to 15 min stimulation with MCh; each blue bar is the average final C-sweat volume in response to 30 min of β-adrenergic cocktail. (B) C-sweat/M-sweat ratios for each test. (C) Gland-by-gland correlation of mean C/M sweat ratios, expressed as a% of the WT average, vs. the mean M-sweat response. Each symbol represents mean values for a single gland based on ∼4 tests. Responses for this subject were at the low end of the control range. (D) Time course of C-sweating in 11 responsive glands selected to cover the range of C-sweat secretion rates.

As stated in methods, the comparison C/M ratio for the CF subjects in this study was the mean C/M ratio of 0.2651 obtained from 6 healthy control subjects in our initial report of the bioassay [10]. As an additional, contemporaneous control, we tested a female healthy control 4 times using an identical protocol. Summary results are shown in **Fig. 10** and **Table 2**). For this subject the ROI contained 51 identified glands. The mean M-sweat final volume for the 15 min test period was 33.9±1.8 nl^−gland^ (51 glands, 4 tests); means for individual tests were: 35.9±2.0, 37.0±2.1, 28.8±1.8 and 36.5±3.2 nl^−gland−30 min^ (**Fig. 10A**, open columns). C-sweating was observed for all but one gland in one test. The mean final volume of C-sweat for the 30 min observation period was 10.24±0.77 nl^−gland^; mean values for each of the four trials were: 9.7±0.7, 9.4±0.7 11.5±1.0 and 10.5±0.9 (**Fig 10A**, blue columns). The mean C/M ratio was 0.143±0.005; ratios for each test were: 0.129±0.006, 0.124±0.004, 0.203±0.015 and 0.178±0.016 (**Fig. 10B** **).** **Fig. 10C** plots the gland-by-gland comparison of C-sweating (as C/M ratio as a percent of the previously determined WT mean) vs. M-sweat. The overall C/M ratio for this control subject was 54±0.02% of the WT average, which is at the lower end of the range for the WT subjects (range: 52–142%). A significantly positive trend between the C/M ratio and M-sweat volumes was also observed in this WT subject; r = 0.55, p = 0.00002. The response time course for C-sweating of 11 selected glands from one test is shown in **Fig. 10D**; unlike the CF subjects, all glands had produced measureable C-sweat volumes by 12 min post-injection.

### Individual differences across subjects and sensitivity of the C/M ratio to sweat gland secretory capacity

**Table 1** shows summary results for all 6 G551D CF subjects; **Table 2** has the summary results for the other subjects.

**Figure 11:**
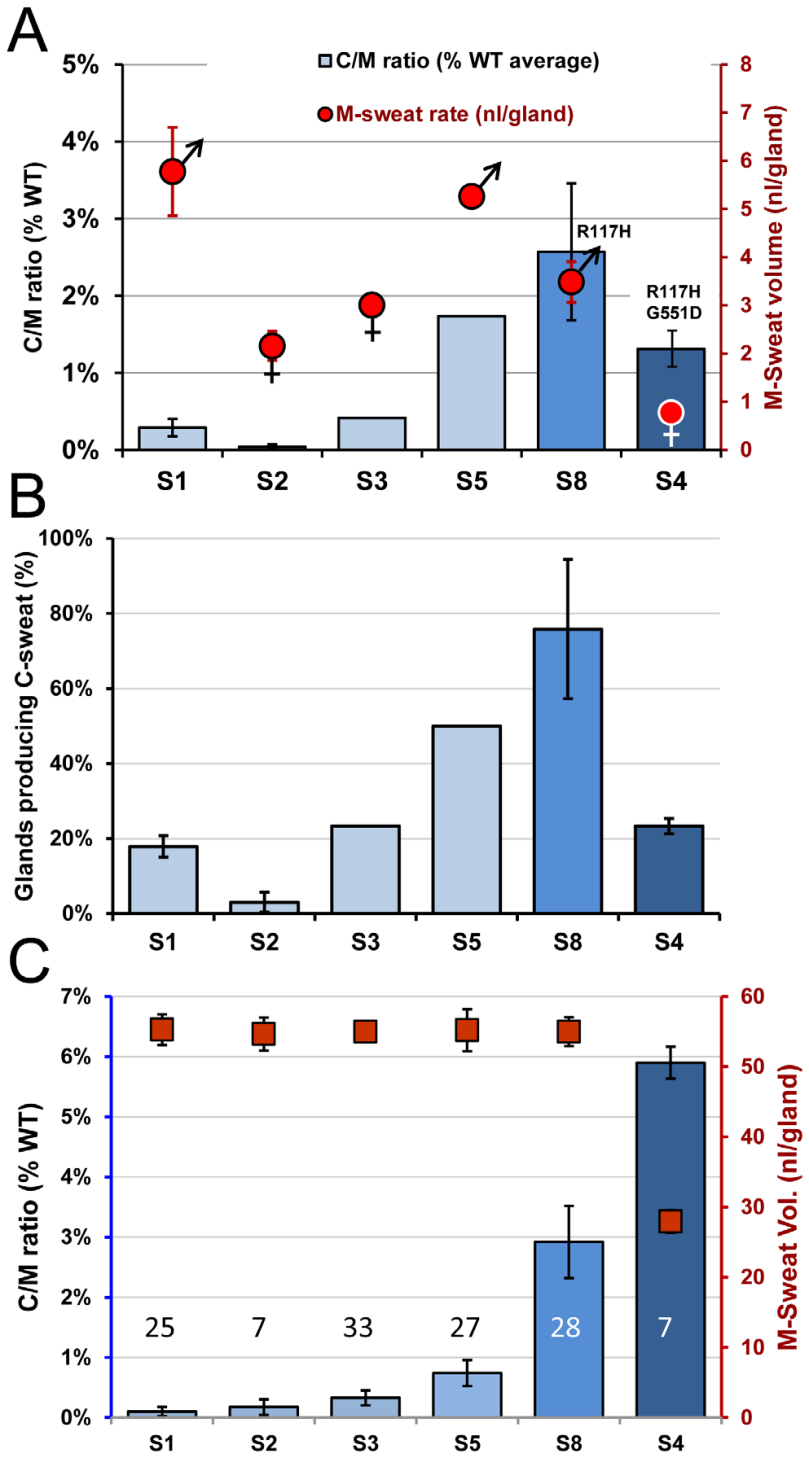
Summary of CFTR-dependent sweat secretion for six CF subjects (+) ivacaftor. (A) Columns (left axis) show C-sweat/M-sweat ratios (mean ± SEM) for each subject, expressed as% of mean WT value. Filled red gender signs (right axis) show M-sweat rates for each subject in the presence of ivacaftor (Mean ± SD). Error bars for S1, S4 and S8 are based on 3 tests, S2 on 2 tests. S1-S3 and S5 each has one G551D mutation, S8 has R117H-5T, and S4 has both. (B) Percentage of glands producing C-sweat in each subject (Mean ± S.D). For healthy control and CF heterozygote (carrier) subjects this value is 97–99±3% [10]. (C) C/M ratios (+) ivacaftor at equivalent M-sweat rates. Filled red squares (right axis) show M-sweat rates (mean ± S.D) for a sample of 7–33 glands from each subject chosen to give a mean M-sweat volume of ∼55 nl^−gland^ ( = 3.67 nl^−min−gland^). Columns (left axis) show C/M ratios (mean ± SEM), expressed as% of mean WT value for the same sample of glands. Numbers indicate sample size for each subject. S4 had the fewest glands and the lowest M-sweat rates, which prevented matching her sample to the others.

**Fig. 11** provides summary data and analyses for all 6 CF subjects tested (+) ivacaftor. **Fig. 11A** plots the mean C/M ratios (left axis, blue columns) and M-sweat rates (right axis, red symbols that also indicate the subject's gender). Mean single gland M-sweat rates (probably determined mainly by gland size) varied widely and were larger for males. (The number [density] of glands also varies widely among individuals, but because we use individual glands as the units of analysis, this factor was not confounded with secretion rate, as it is in most sweat assays.) **Fig. 11B** plots the percentage of identified glands from each subject that produced measurable volumes of C-sweat. The G551D subjects differed significantly from one another in the proportion of glands that produced measureable C-sweat (ANOVA, F = 54.8, *p* = 0.001). **Fig. 11C** plots the C/M ratios after equating M-sweat rates. Because C-sweating is jointly dependent on M-sweating and CFTR function, we isolated CFTR's contribution by selecting a subset of glands from each subject such that the average M-sweat rates for the samples were nearly equivalent (**Fig. 11C**, right axis, red squares). The corresponding C/M ratios for these matched samples of glands (**Fig. 11C**, left axis, blue columns,) show that G551D subjects still differ widely: e.g. S5's ratio is ∼7-fold >S1's. Even so, the differences within G551D subjects are overshadowed by the much higher C/M ratio seen in R117H-5T subject 8, and in S4, who, with two responsive mutations, had the highest C/M ratio after partially correcting for M-sweat rate.

**Figure 12:**
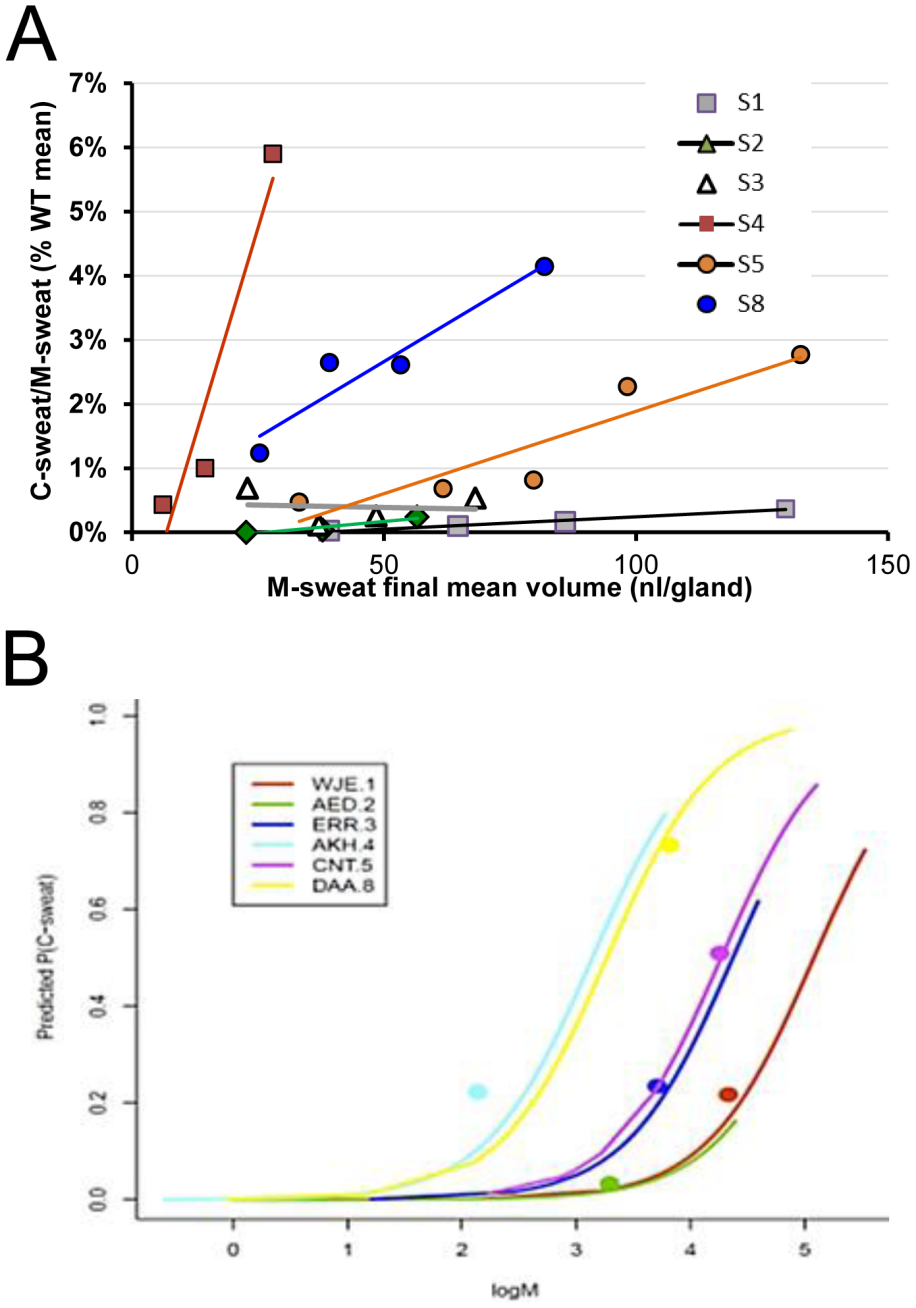
C-sweat values as a function of M-sweat rates. (A) Summary data showing dependence of C/M ratios to M-sweat rates for all 6 subjects tested (+) ivacaftor. For clarity, each subject's glands, glands were rank ordered according to their M-sweat rates and then divided into 3–5 bins according to their M-sweat rates (mean of 16.6 glands per bin). Each point plots the average M-sweat response on the x-axis and the average C/M ratio as a% of the WT mean on the y-axis for each bin; lines are linear fits. Each gland was measured 1–3 times, and the average number of glands included in each bin was 16.6 (range 5–26 glands). C/M vs. M-sweat correlations were significant for all subjects except S3. (B) C-sweat response is coded as 1, if some C-sweat is observed on a test, and 0, otherwise; and P(C-sweat) denotes the probability that C-sweat is observed. Then P(C-sweat) is also the average of the recoded 0/1 C-sweat response. We predicted P(C-sweat) as a function of log M-sweat using the combined data from all 6 CF subjects tested (+) ivacaftor. A logistic mixed model analysis showed that CF patients differed in their overall P(C-sweat) levels, but not in the within-patient effect of M-sweat response on P(C-sweat) across glands (p>.57), and that the average across patients of the within-patient effect of M-sweat was significantly positive (b = 2.14, p<.0001). The predicted P(C-sweat) levels are plotted for each patient. See methods and Appendix S1.

**Figure 13:**
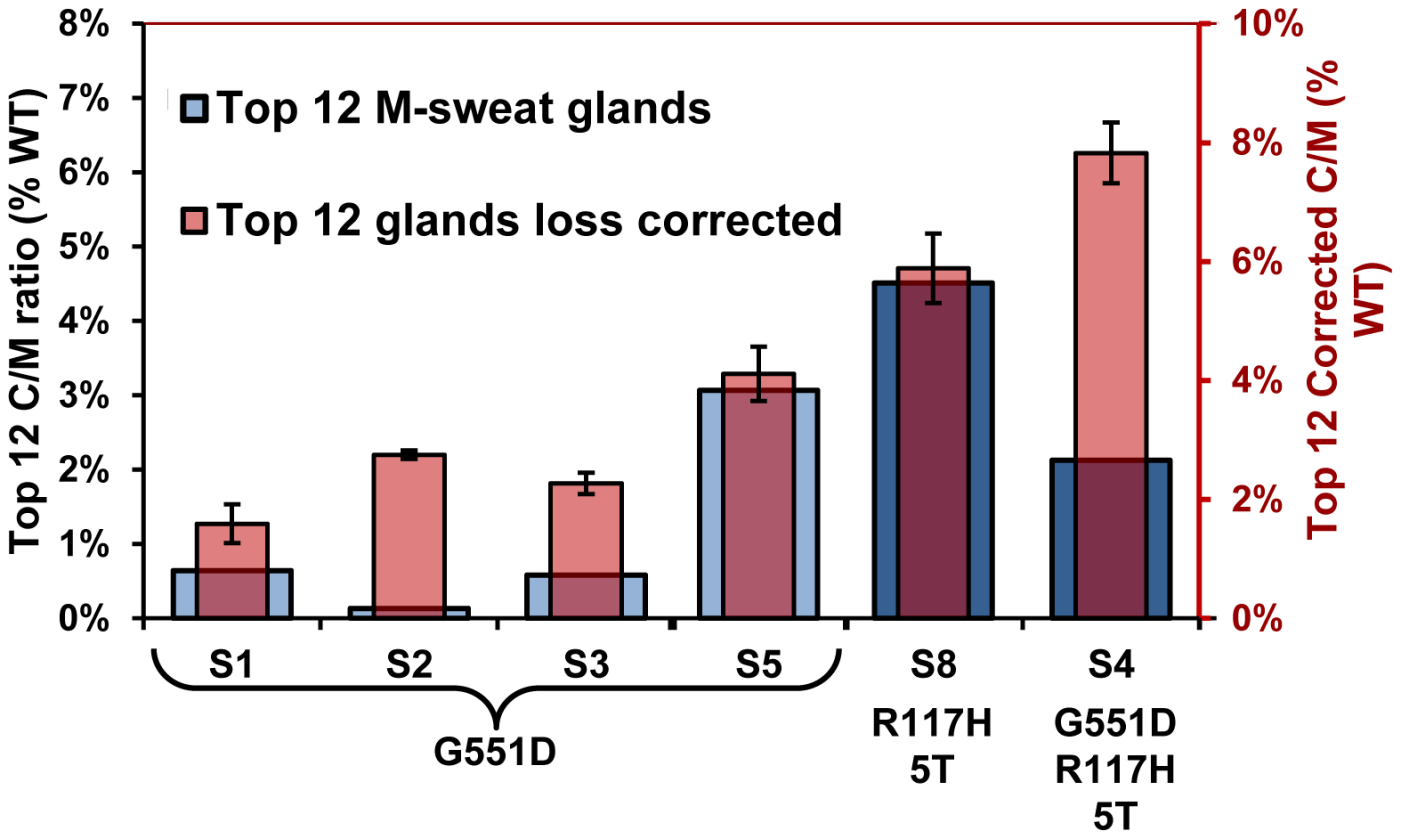
Loss-reduced and loss-corrected estimates of CFTR function in CF subjects (+) ivacaftor. Each blue column (left axis) shows mean C/M ratios (mean ± SEM) for the 12 glands with the highest M-sweat rates for each subject; higher sweat rates reduce C-sweat losses. Each transparent red column (right axis) shows the corrected C/M ratios (mean ± SEM) for the 12 fastest M-secreting glands, obtained by adding 0.029 nl^−min−gland^ to the observed C-sweat secretion rates (see text).

A consistent result of this study is that the C/M ratio in subjects with low C-sweat rates is positively correlated with M-sweat rates (**Figs. 2C** **,** **3C** **,** **4A** **,** **5A** **,** **8D** and **9C** and **10A**). This relationship is summarized for all subjects in **Fig. 12A**. It was previously hypothesized that the positive correlation resulted from at least two subtractive processes: physical capacitance (filling of gland lumen before sweat emerges) and absorption of sweat volume in the duct [10]. These have negligible consequences at high sweat rates, but become progressively evident at lower sweat rates. If this hypothesis is correct, it follows that the larger values for each subject, rather than the average for all glands, should more accurately quantify CFTR function. Accordingly, **Fig. 13** (blue columns, left axis) plots for each subject the mean C/M (% WT) of the 12 glands having the highest M-sweat rates, which yielded C/M ratios from <1%–4.5% of the mean WT ratio. The red columns in **Fig. 13** are based on an additional correction that incorporates data from single channel measurements (see discussion).

## Discussion

Our first goal in this study was to determine if this bioassay could detect improved CFTR function in CF subjects with G551D or R117H-5T mutations who were taking ivacaftor. We knew before starting that ivacaftor increases G551D CFTR function sufficiently to benefit health [4], [5]. Therefore, the positive results with these subjects validate the assay rather than the compound. But no clinical data were available on ivacaftor treated R117H-5T subjects, so our finding of a strong ivacaftor effect for subjects with this compound allele provides *in vivo* support for the prediction that ivacaftor will be therapeutic in such patients [2].

A second goal was to determine if the assay could detect individual differences among the subjects. In fact, we observed large (∼6-fold), inter-subject differences in C-sweat responses among the four G551D subjects taking ivacaftor, after correcting for M-sweat rates (**Fig. 11C**). Individual differences could arise from different G551D-CFTR expression levels, differences in transport processes affecting CFTR-dependent sweat secretion, drug pharmacokinetics/tissue distribution, different responses of the in trans F508del allele, and compliance. Such differences are expected to have clinical implications. We were blinded to results from clinical studies of these subjects, but it will be possible to compare the two data sets in the future.

A third goal was to determine if the assay could quantify the extent to which ivacaftor restored function to G551D and R117H-5T CFTR. We hypothesized that primary C-sweat (as elaborated in the secretory coil) provides an accurate readout of CFTR function [10], and that some primary secretion was occurring in most treated glands. However, because subtractive processes reduce the volume of primary C-sweat, uncorrected C-sweating underestimates CFTR function. Therefore, we used independent evidence about channel number and *P_O_* to correct for the losses.

**Figure 14:**
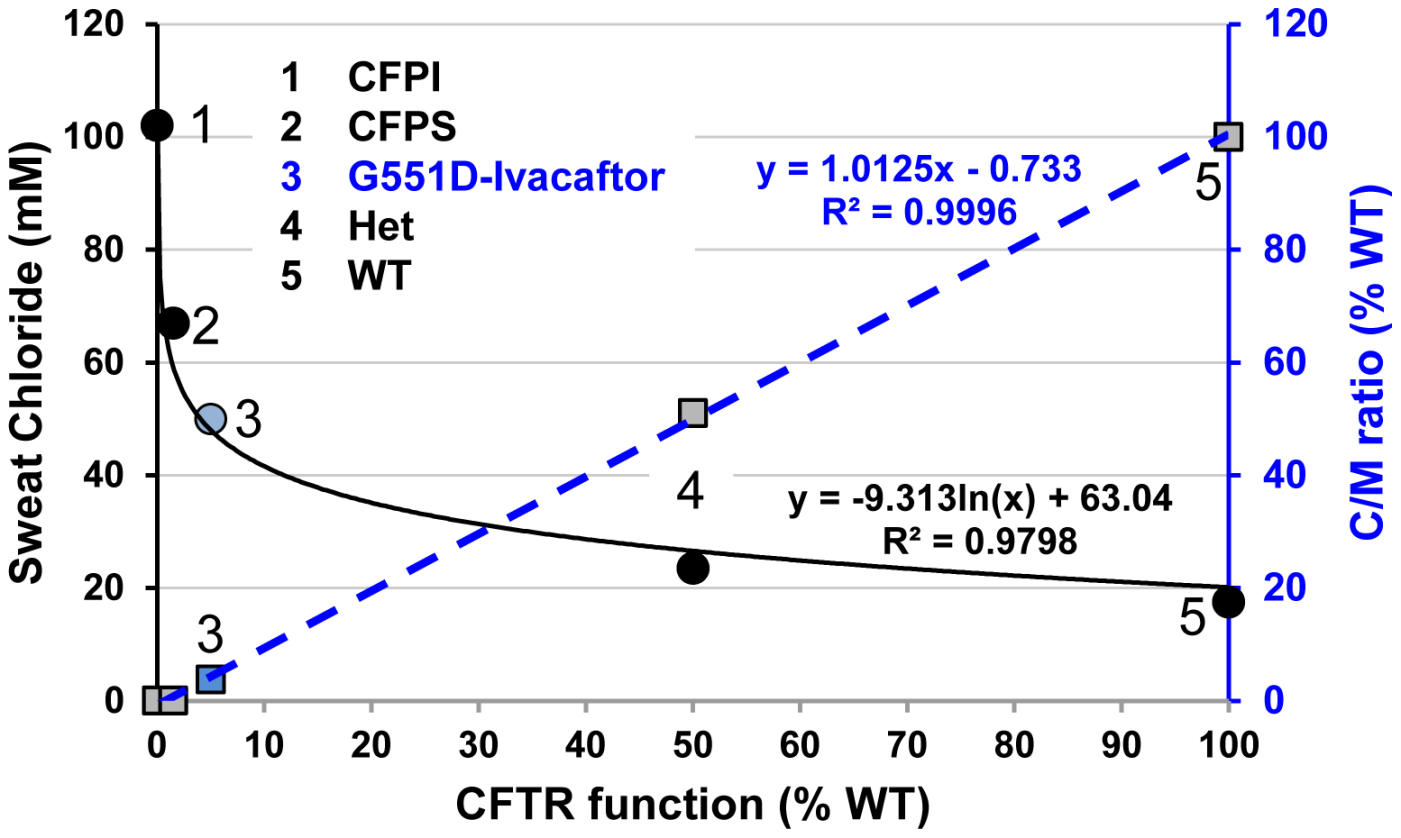
Two measures of CFTR function in the sweat gland. The sweat chloride concentration (left y axis,) is plotted (solid line) against CFTR function as a% of average WT values, plotted on the x axis. Sweat chloride is an inverse logarithmic function of CFTR function (n *P_O_* γ) and hence is most sensitive at the lowest levels of CFTR function. The C-sweat/M-sweat ratio as% WT (right axis), is plotted (dashed line) against CFTR function as a% of average WT values, plotted on the x axis. The C/M ratio provides a direct readout of CFTR function and is linear over most of the range; the loss-reduced and loss-corrected values extend the linear range close to zero CFTR function. Sweat chloride point 3 is from ref. [5], the rest are from ref. [38]. C/M point 3 is from present paper, other points are from ref. [10]. CFTR values 0, 50 and 100 are defined, 1.5% and 5% function assigned by patch clamp and mRNA measures.

CF is caused by reductions in apical membrane conductance mediated by CFTR, which in turn is a function of channel number, open probability, and single channel conductance: G_CFTR_ = n*P_O_*γ. The *P_O_* of WT CFTR is ∼0.4, e.g. ref. [2]. When considering channel number (n), it is necessary to take account of wide variations arising especially from the length of a polythymidine tract in intron 8 [13]. This tract is found as 3 variable repeat alleles of 9T, 7T or 5T, which in the non-CF population exist in proportions of ∼9%, 80% and 11% respectively [20]. The tract length influences splicing of exon 9 such that as tract length shortens, exon 9 skipping increases, resulting in less functional CFTR [13]. With the 5T allele, only ∼10% of full-length, functional CFTR is produced, *vs*. 50–100% with 7T and >95% with 9T [13]. Furthermore, a variable TG repeat also affects the number of functional CFTR transcripts [21]. Thus, healthy controls have a wide range of CFTR (n) and hence CFTR function. Importantly, this variation is seldom considered when assessing the operation of most organs with *in vivo* assays, because CFTR function is logarithmically related to most outputs (e.g. sweat chloride, **Fig. 14**) and hence is not rate limiting until it approaches zero (which is why CF is a recessive disease). However, CFTR *is* rate-limiting for C-sweating, which accounts for its linear output [10], [22] and (**Fig. 14**). Thus, variation in CFTR (n) is expected to be expressed quantitatively in the C/M sweat ratio. Indeed, the C/M ratio varied 3-fold among 6 healthy controls tested with this method [10] and varied 4-fold among 19 controls in the original study of C/M ratios using a different method [22]. In both studies CF heterozygotes showed a similar dispersion around a mean C/M value half that of the healthy controls [10], [22]. On average, healthy control subjects have 70% full length transcripts [13], which we used to set the average WT value for n in the following estimates of CFTR function, i.e. we assume that ∼70% transcripts gave the mean C/M ratio of 0.265 for healthy controls [10]. With this background, we first consider results for the R117H-5T subject.

### Quantifying R117H-5T CFTR function in vivo and in vitro

R117H-5T CFTR retains partial function and confers pancreatic sufficiency when *trans* to a severe allele. Studies of heterologously expressed CFTR channels by Sheppard and colleagues [12] found that the whole-cell Cl^−^ conductance of R117H was reduced to 15% of WT, while single channel analysis found that both *P_O_* and γ were reduced predicting ∼24% WT CFTR channel function. In a male and a female each having R117H/R117H on a 7T/7T background, all clinical indices were normal except for CBAVD in the male, electrophysiological measurements of nasal and intestinal epithelia were also normal, and sweat chloride values were 34 mM for the male and 42 mM for the female [23]. However, when R117H-CFTR is in *cis* with a 5T allele, n = 0.1/0.7 = 14% WT, reducing R117H-5T function to 2.1–3.7% of WT function. If the other allele is nonfunctional, as it is in S8, the expected residual function will be 1.1%–1.85% of WT (average 1.5%). Remarkably then, <2% of CFTR function, if also found in the pancreas, appears to be sufficient to confer pancreatic sufficiency and to reduce sweat chloride levels from 100 to ∼80 mM [24].

To estimate the amount of primary sweat in S8 that was lost in the (−) ivacaftor condition, we assigned a CFTR functional level = 1.5% WT to S8, based on the above analysis of R117H-5T function [12], [13]. For S8's 12 glands with highest M-sweat rates, the predicted rate of C-sweating, if he were a WT subject, would be 1.59 nl^−min−gland^, and 1.5% of that value = 0.024 nl^−min−gland^. However, we observed only 0.001 nl^−min−gland^, leading to a loss estimate of 0.023 nl^−min−gland^. If ivacaftor restored normal *P_O_* to R117H and left n and γ unchanged, it would increase R117H-5T CFTR function equivalent to 5T function on 1 allele, or 7% of WT. The C/M ratio for the top 12 glands of S8 (+) ivacaftor was 4.51% of WT (left axis, blue column, **Fig. 12B**). Correcting for the loss estimate increased the C/M ratio to 6.1% of WT (right axis, red column, **Fig. 12B**). Thus, in this R117H-5T subject, functional estimates based on patch-clamp [12] and mRNA transcript analysis [13] converged with those of based on C/M-sweat bubble analysis.

### Quantifying G551D CFTR function in vivo and in vitro

The G551D mutation severely decreases *P_O_* to near zero (∼0.004) without a known effect on n or γ [15]. The calculations used above predict that this level of function is invisible to the sweat bubble test, consistent with the lack of any observed sweating in the 4 G551D subjects tested (−) ivacaftor. The P_O_ of G551D-CFTR (+)-ivacaftor has been measured as 0.04 [14] and 0.12 [2] or 10–30% of the WT value of 0.4. Subjects S1–S3 and S5 each have one G551D and one (nonfunctional) F508del allele, so their ivacaftor-potentiated CFTR function should have been half of those values or 5–15% of WT. C/M ratios from the top 12 M-sweat rate glands (**Fig. 12A, B**) gave values of 0.24% to 2.77%, and correcting for the loss estimate of 0.023 nl^−min−gland^ gave 1.56% for S1 to 4.08% for S5. The highest value is similar to the estimate of ∼5% CFTR function predicted from the ivacaftor-potentiated *P_O_* value of 0.04 [**14**]. Based on all the data and on comparisons with the ivacaftor-treated R117H subject, we estimate ivacaftor-potentiated CFTR function in G551D/F508del subjects to be <5% WT.

Subject S4 has mutations R117H-5T and G551D. In the (+) ivacaftor condition, the data from patch clamp and transcript analysis predicts CFTR function of 2.6–5% function for R117H-5T, and up to 5% for G551D, or up to 10% function for the combination. The corrected C/M value for this subject was 7.7%, the highest value observed, consistent with an additive effect of ivacaftor acting on both mutations.

### Does ivacaftor increase M-sweating?

The present experiments do not answer that question. Two of 3 subjects where M-sweating was tested (+/−) ivacaftor did not show a change. The increased M-sweating seen in S1 (+) ivacaftor occurred in tests that were run over a 6 month period, and two (−) ivacaftor tests in winter months gave lower values than any other tests. There was no opportunity to test again off-drug. M-sweating has been considered to be CFTR-independent because of its persistence in CF subjects [16], [17], but CFTR contributes to cholinergically-mediated fluid secretion in several tissues and species [25], [26], [27], [28], [29], [30], [31]. Stimulation of M3 muscarinic receptors strongly activates CFTR when both are expressed in BHK cells [32], and apical UTP activates CFTR in primary human airway cells [33]. It is possible that CFTR is activated during M-sweating, but its contribution may be masked by a high apical conductance conferred by CaCC channels.

### Limitations

Four limitations specific to the present study are that it was done open label, with a small number of subjects, the results could not be correlated with clinical or sweat chloride data because of ongoing studies, and only two subjects were tested with triplicate (±) ivacaftor tests. The inaccessibility of subjects for repeated (±) testing was our main concern, because the assay is well suited for n-of-1 studies where each subject (and each gland) serves as its own control [34], [35], [36], [37]. It will be important to carry out more n-of-1 studies with ivacaftor in which this assay is one component of the assessment, and to establish the assay in other laboratories. Because of large and consistent individual differences in measurements of healthy controls, it will be important to expand the number control subjects and to correlate their results with genotype, sweat chloride, and CFTR transcript analysis.

## Conclusions

Multiple measures of C-sweating and M-sweating from individual glands revealed that oral dosing with ivacaftor improved β-adrenergic cocktail-stimulated, CFTR-dependent sweating (C-sweating) in CF subjects with G551D or R117H-5T mutations. The C/M ratio across all measured glands was smaller than predicted based on patch clamp/transcript analyses. We hypothesize that this resulted from a proportionally larger loss of sweat volume via ductal absorption and gland capacitance at lower sweat rates. After correcting for these losses, estimated CFTR function was 2.66±1.06% of WT for the 4 G551D subjects, 6.1% for the R117H-5T subject, and 7.7% for the subject with both mutations. The finding that CFTR-dependent sweat secretion was greater in R117H-5T than in G551D subjects supports prior work suggesting that ivacaftor will be clinically useful for subjects with the R117H-5T mutation [6]. In sum, the corrected *in vivo* estimates of restored CFTR function overlap estimates based on patch clamp data, and suggest that excellent clinical responses can be expected by restoring less than 10% of CFTR function.

## Supporting Information

Appendix S1 (DOCX)
