# Supplementary material for: A Little CFTR Goes a Long Way: CFTR-Dependent Sweat Secretion from G551D and R117H-5T Cystic Fibrosis Subjects Taking Ivacaftor
Source: PLoS One. 2014 Feb 10;9(2):e88564. doi: 10.1371/journal.pone.0088564 (PMC3919757; doi:10.1371/journal.pone.0088564)
Supplement: Appendix S1 — (DOCX) [file pone.0088564.s001.docx]

**Appendix S1**

*Mixed models analysis of C- and M- sweating (+) and (-) ivacaftor.*

The ratio of the average rates of M- and C-sweat produced per gland (averaged over 15 min for M-sweat and 30 min for C-sweat) is the main index of sweat production, and we use it here to estimate the proportion of CFTR function in various conditions relative to a sample of healthy controls. However, for CF subjects, virtually no glands produced C-sweat in the (-) ivacaftor condition, and the majority still failed to produce measurable C-sweat (+) ivacaftor. We assume that glands sometimes secrete amounts of sweat that fail to exceed the threshold resulting from subtractive processes [[10](#_ENREF_10)]. To assess this assumption, we dichotomized the C-sweat response as 0, if there is no observed secretion, and 1, otherwise. With this recoding, the average recoded C-sweat response is simply the *probability* that a gland produces C-sweat on a test, and we use this probability as an alternative index of C-sweat production.

We applied this approach as follows:

One R117H-5T and two G551D subjects were tested using an n-of-1 paradigm in which, for each subject, tests on multiple glands were given (-) and (+) ivacaftor. For the G551D subjects, no glands responded in the (-) ivacaftor condition and some glands responded (+) ivacaftor. That qualitative difference, while gratifying, poses difficulties for conventional statistical tests and led to the following approach to pre-processing the data. Each test in which a gland secretes C-sweat was represented by ‘1’, regardless of the volume of sweat produced, and a non-secreting test was represented by a ‘0’. The difficulty we faced was that logistic mixed models regression on the raw data containing only ‘0’s in the (-) ivacaftor condition yielded unreliable estimates of the condition (i.e., ivacaftor) effect. However, by using a mixed model with appropriate parameter values to simulate various data sets, reliable parameter estimates could be obtained with as few as a single ‘1’ in the (-) ivacaftor condition. Therefore, starting with S1’s data, we sought a principled method for assigning a ‘1’ to as few gland tests as possible in the (-) condition.

All glands of S1 that produced C-sweat on all three of the (+) ivacaftor trials were identified. There were four such ‘active’ glands out of a total of 62, accounting for a total of twelve [4(glands) times 3(tests)] ‘0’s in the (-) ivacaftor condition. We created twelve new data sets by sequentially converting to ‘1’ exactly one of the twelve ‘0’s in the (-) ivacaftor condition. The parameter estimates from the mixed models analysis of each of these minimally perturbed data sets were noted, and then were averaged across the 12 replications. We report the modal value of the estimate of the condition effect as a lower bound for the true value.

The data for S2, with up to 2 tests in each of the (-) and (+) ivacaftor conditions, were treated in a similar way. For this subject, only one out of 91 glands produced C-sweat on both of the (+) ivacaftor trials. We created a new data set by converting to ‘1’ exactly one of the two ‘0’s from this ‘active’ gland in the (-) ivacaftor condition, and the parameter estimate from the analysis of this minimally perturbed data set is reported.

For the CF subject with R117H-5T (S8), ivacaftor also produced an unequivocal increase in CFTR-dependent secretion. However, in this pancreatic sufficient subject, 1 of 53 glands in the ROI produced C-sweat in each of 3 tests in the (-) ivacaftor condition. This same gland produced C-sweat also in each of 5 tests in the (+) ivacaftor condition, and it was one of 16 glands to do so. This data set, therefore, is typical of our simulated data sets in which (a) very few glands produce C-sweat in the (-) ivacaftor condition, and (b) these are the glands that are also very productive in the (+) ivacaftor condition. As was found in our simulated data sets, S8’s raw data also yielded reliable parameter estimates, and these are reported below.

We explored the validity of our estimates of the condition effect by seeing if they varied in expected ways as larger perturbations were made of the data for these 3 subjects.

**S1**: Using the data from the four ‘active’ glands of S1, we created 3 other data sets with two, three and four ‘1’s in the (-) ivacaftor condition, such that no gland had more than one ‘1’. As expected, the estimate of the condition effect decreased as the number of ‘1’s increased. Measured on a log-odds scale, the effect was 4.86, 4.23, 3.83 and 3.54, when the number of ‘1’s was 1, 2, 3 and 4, respectively.

**S2**: Using S2’s data, we created 3 other data sets using the data from ‘mildly active’ glands that produced C-sweat on 1 of the 2 (+) ivacaftor trials. Considering the test results from the first ‘mildly active’ gland, we formed the first of these data sets by converting to ‘1’ one of the two ‘0’s in the (-) ivacaftor condition *and* the ‘0’ in the (+) ivacaftor condition. Then starting with this data set, we formed the second data set by doing the same to the results for the second ‘mildly active’ gland; and we repeated this exercise with the third ‘mildly active’ gland to generate the third data set. The results from the minimally perturbed data set were consistent with those from these latter three data sets, inasmuch as the condition effect was not statistically significant (p > .13) in any of the 4 analyses.

**S8**: Finally, we minimally perturbed the data from the 16 ‘active’ glands in S8’s data by converting one ‘0’ to ‘1’ in the (-) ivacaftor condition. For all 15 such perturbations, the estimate of the condition effect was 5.88, less than the estimate of 6.19 yielded by the raw data.

Taken together, these calculations provide some justification for the perturbation method we used, and for our interpretation of the parameter estimate from the minimally perturbed data set as a lower bound for the true value of the condition effect. Note that this method is useful for testing statistical significance when many glands do not respond, but becomes insensitive as the response rate increases and P(C-sweat) approaches 1. For example, WT and HZ both have P(C-sweat) ≈ 1, but C/M ratios of 1 and 0.5 respectively. The upper bound for using this method is a function of CFTR functional levels and gland secretory capacity (M-sweat rate).

We now summarize the questions to be addressed below. Each subject furnishes a large number of glands, each of which is measured on a number of occasions (labeled as ‘week’) for M-sweat levels and C-sweat levels in the (-) Ivacaftor condition and/or in the (+) Ivacaftor condition. ‘Gland’ is treated as a random effect, with glands possibly varying in the typical *amount* of sweat they produce (i.e., in the corresponding ‘intercept’ of the mixed model), and in the *effect* of ivacaftor on the amount of sweat produced (i.e., in the corresponding ‘slope’). Measurement occasion (or ‘week’) is also treated as a random effect that adds the same random amount to all sweat measurements that week (i.e., with varying ‘intercept’). Ivacaftor condition ((-) versus (+)) is included as a fixed effect in the mixed model.

1. Is the mean amount of M-sweat produced on a test the same in the (-) Ivacaftor and (+) Ivacaftor conditions?

2. Is the probability that a gland produces C-sweat on a test, P(C-sweat), the same in the (-) Ivacaftor and (+) Ivacaftor conditions?

3. Considering only the (+) Ivacaftor condition, in which P(C-sweat) is not negligible, does the amount of M-sweat produced by a gland predict P(C-sweat) for that gland?

4. In examining each of these three questions, what is an appropriate model for the random effects of *gland* and *week*? Do glands differ significantly in their typical levels of sweat production, and in their sensitivity to Ivacaftor? Are the differences in sweat production across weeks statistically significant?

5. What are the similarities and differences among CF patients in the prediction of level of CFTR function using M-sweat levels as the predictor?

*Regression analysis of responses for three subjects tested (±) ivacaftor.*

In these analyses we use the prediction of P(C-sweat) from M-sweat (+) ivacaftor as the index of CFTR function. We dichotomized C-sweat, so that the average of the recoded response = P(C-sweat)*1 + [1 - P(C-sweat)]*0 = P(C-sweat).

**Subject 1** (**S1**, male, F508del/G551D) was tested 3 times in each condition. We developed a prediction equation based on the gland’s M-sweat response (+) ivacaftor, as discussed above. If the significant differences in P(C-sweat) across glands is due mainly to factors, e.g., gland size, that are reflected in the M-sweat response (+) ivacaftor, then one would expect that, when M-sweat response is included as a predictor, ‘gland’ would no longer be significant as a random effect. Similarly, to the extent that differences among weeks are reflected in the M-sweat response, ‘week’ would no longer be significant as a random effect. A logistic mixed model analysis showed that M-sweat response (on a log scale) was a significant predictor of P(C-sweat) (b = 2.43, p < .0001), in the (+) ivacaftor condition. Neither ‘gland’ nor ‘week’ was significant in the (+) ivacaftor condition. This latter finding suggests that the effects of ‘gland’ and ‘week’ on CFTR function are mediated by the CFTR-independent M-sweat response. On the log scale, M-sweat levels vary from 1.045 to 5.527, and the corresponding probabilities of a C-sweat response are .00006 and .23 (see discussion). See Appendix Fig. 1.

**Subject S2** (**S2,** female, F508del/G551D) was tested twice in each condition. Glands differed significantly in their typical M-sweat rates (p < .0001), and in their sensitivity to ivacaftor (p < .0001). Indeed, these differences in sensitivity likely account for the absence of a main effect of ivacaftor. Ivacaftor increased M-sweat secretion for some glands (*N* = 56) and decreases it for other glands (*N* = 33), yielding a significant ivacaftor-by-gland interaction but no ivacaftor main effect. There was also marginally significant variation in average M-sweat rates across weeks (p = .051). The statistical significance of these differences indicates the sensitivity of the multi-gland test, but their biological significance is questionable. Their most likely source lies in variable agonist delivery that is unavoidable with intradermal injections. In predicting P(C-sweat) from M-sweat response (+) ivacaftor, a logistic mixed model analysis showed that M-sweat response (on a log scale) was a significant predictor of P(C-sweat) (b = 3.88, p < .05).

**Subject 8** (**S8**, I507del/R117H-5T) was tested 3 times (-) and 5 times (+) ivacaftor. With dichotomized C-sweat responses, a logistic mixed model analysis revealed a significant effect of ivacaftor [P(C-sweat) estimates were .8065 and .0085 in the (+) and (-) ivacaftor conditions, respectively, p < .0001]. Glands (p < .0001) and weeks (p < .005), differed significantly in their probability of a C-sweat response. S8’s M-sweat values did not differ between (+) and (-) ivacaftor. Glands differed significantly from one another in their typical M-sweat rates (p < .0001), but not in their sensitivity to ivacaftor (p > .45). There was also significant variation in average M-sweat rates across weeks (p < .0001, data not shown), probably reflecting variations in agonist delivery. In predicting P(C-sweat) from M-sweat response (+) ivacaftor, a logistic mixed model analysis showed that M-sweat response (on a log scale) was a significant predictor of P(C-sweat) (b = 2.00, p < .0001), again suggesting that the effects of ‘gland’ and ‘week’ on CFTR function are mediated by the CFTR-independent M-sweat response. On the log scale, M-sweat levels vary from -0.064 to 4.998, and the corresponding probabilities of a C-sweat response are 0.0013 and 0.9711.

*The effect of M-sweat on P(C-sweat) is approximately constant across all six subjects tested (+) ivacaftor.*

Our findings that, for the six patients tested (+) ivacaftor, estimates of the within-patient effect of M-sweat on P(C-sweat) lay between 2 and 4 led us to examine whether the differences among patients in this effect were not significantly different from 0. Accordingly, we collated the data from all six patients into one data set, and then applied logistic mixed models in which log(M-sweat) was a fixed-effects predictor, and ‘patient’ was a random-effects predictor. We compared two models, namely, one in which patients varied only in their typical P(C-sweat) level, and the other in which patients varied in their typical P(C-sweat) level and in the within-patient effect of M-sweat on P(C-sweat). We found that the second model did not provide a significantly better fit to the data than the first model (p > .57). Further, the average across patients of the within-patient effect of M-sweat was significantly positive (b = 2.14, p < .0001). The predicted P(C-sweat) levels are plotted for each patient in Fig. 12B.
